# The impact of different forms of exercise on circulating endothelial progenitor cells in cardiovascular and metabolic disease

**DOI:** 10.1007/s00421-021-04876-1

**Published:** 2022-01-12

**Authors:** Panagiotis Ferentinos, Costas Tsakirides, Michelle Swainson, Adam Davison, Marrissa Martyn-St James, Theocharis Ispoglou

**Affiliations:** 1 Carnegie School of Sport, Leeds Beckett University, Leeds, UK; 2 Lancaster Medical School, Faculty of Health and Medicine, Lancaster University, Lancaster, UK; 3 Flow Cytometry Facility, Leeds Institute of Cancer and Pathology St James’s University Hospital, University of Leeds, Leeds, UK; 4 Cytec Biosciences B.V, Amsterdam, The Netherlands; 5 School of Health and Related Research, University of Sheffield, Sheffield, UK

**Keywords:** Endothelial progenitor cells, Exercise, Cardiometabolic health, Cardiovascular disease, Vascular health, Flow cytometry, EPC mobilisation, Resistance exercise, High intensity interval training, Moderate intensity continuous training, Aerobic training

## Abstract

Circulating endothelial progenitor cells (EPCs) contribute to vascular repair and their monitoring could have prognostic clinical value. Exercise is often prescribed for the management of cardiometabolic diseases, however, it is not fully understood how it regulates EPCs. Objectives: to systematically examine the acute and chronic effects of different exercise modalities on circulating EPCs in patients with cardiovascular and metabolic disease**.** Methods: Preferred Reporting Items for Systematic Reviews and Meta-analyses guidelines were followed. Results: six electronic databases and reference lists of eligible studies were searched to April 2021. Thirty-six trials met the inclusion criteria including 1731 participants. Acute trials: in chronic heart failure (CHF), EPC mobilisation was acutely increased after high intensity interval or moderate intensity continuous exercise training, while findings were inconclusive after a cardiopulmonary cycling exercise test. Maximal exercise tests acutely increased EPCs in ischaemic or revascularized coronary artery disease (CAD) patients. In peripheral arterial disease (PAD), EPC levels increased up to 24 h post-exercise. In patients with compromised metabolic health, EPC mobilisation was blunted after a single exercise session. Chronic trials: in CHF and acute coronary syndrome, moderate intensity continuous protocols, with or without resistance exercise or calisthenics, increased EPCs irrespective of EPC identification phenotype. Findings were equivocal in CAD regardless of exercise mode, while in severe PAD disease EPCs increased. High intensity interval training increased EPCs in hypertensive metabolic syndrome and heart failure reduced ejection fraction. Conclusion: the clinical condition and exercise modality influence the degree of EPC mobilisation and magnitude of EPC increases in the long term.

**Graphical abstract:**

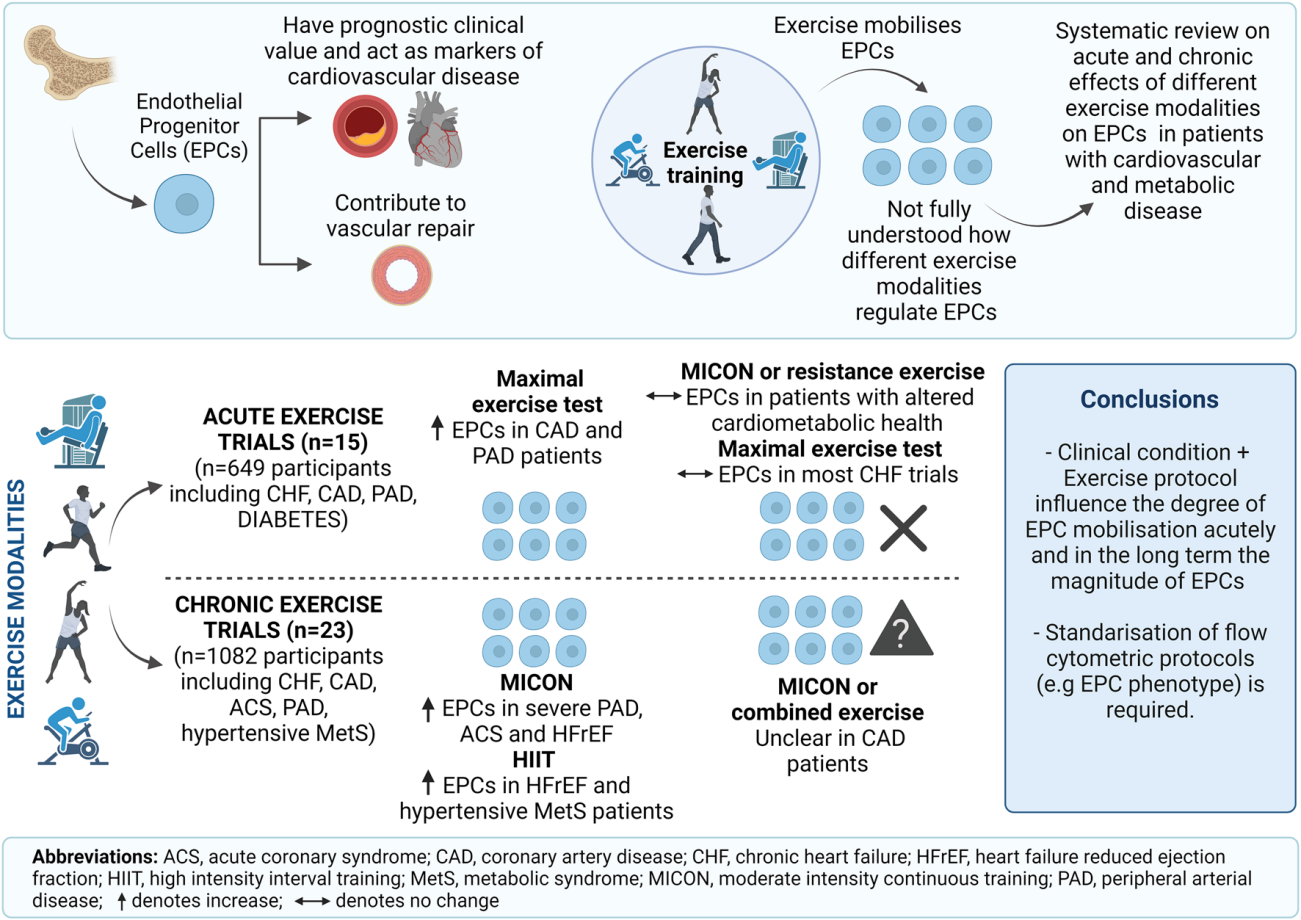

**Supplementary Information:**

The online version contains supplementary material available at 10.1007/s00421-021-04876-1.

## Introduction

The vascular endothelium has many important and diverse functions (Hadi et al. 2005) with regulation of vascular homeostasis being one of the most important ones (Verma and Anderson 2002). Endothelial dysfunction, which is an independent predictor of cardiovascular events, precedes the development of atherosclerosis and cardiovascular disease (CVD) (Widlansky et al. 2003; Gokce et al. 2003). Progression of diseases such as metabolic syndrome and diabetes mellitus (DM) is also strongly associated with the progression of endothelial dysfunction (Fornoni and Raij 2005; Hadi and Suwaidi 2007). At the same time, several cardiovascular risk factors such as ageing, physical inactivity, smoking and hypertension contribute to an exacerbation of endothelial dysfunction as demonstrated by reduced flow-mediated dilatation (FMD) (Steinberg et al. 1996; Black et al. 2009; Amato et al. 2013). Therefore, endothelial repair is essential for the healing of injured endothelium and the prevention of endothelial dysfunction.

Mature endothelial cells have low proliferative capacity (Hristov et al. 2003; Werner and Nickenig 2006) and it was once thought that vasculogenesis, occurred only during embryonic development (Koutroumpi et al. 2012). However, Asahara et al. (1997), following isolation of putative endothelial progenitor cells (EPCs) from human peripheral blood using magnetic bead selection, demonstrated that CD34^+^ cells after seven days of culture started to express markers from endothelial lineage such as CD31, VEGFR-2 and E-Selectin. Those putative EPCs act either directly on the site of injury via migration, proliferation and differentiation or indirectly in a paracrine manner, which in turn results in activation of several pro-angiogenic factors leading to proliferation and migration of pre-existing endothelial cells (Asahara et al. 2011). This critical function of circulating EPCs to promote neovascularisation has brought attention to their role as a surrogate marker of CVD and as a prognostic indicator of cardiovascular events and mortality (Shintani et al. 2001; Vasa et al. 2001; Werner et al. 2005; Lu et al. 2016).

To counterbalance the reduced levels of EPCs and their impaired function in clinical populations, several pharmacological treatments, such as statins and angiotensin converting enzyme inhibitors have been implemented successfully (Sen et al. 2011; Lee and Poh 2014). Moreover, recent studies have reported that lifestyle modifications such as exercise and diet can also increase the number of EPCs and subsequently improve vascular function (Fernandez et al. 2012; De Biase et al. 2013; Maiorino et al. 2017). Accordingly, the latest guidelines for the management of chronic heart failure (CHF) and arterial hypertension recommend regular aerobic exercise as class I and level A of evidence for improvement in symptoms, functional capacity, reduced risk of hospitalisation (McDonagh et al. 2021) and for the reduction of cardiovascular risk and mortality (Williams et al. 2018). Apart from aerobic exercise, the inclusion of resistance exercise 2–3 days per week is also recommended for the management of arterial hypertension (Williams et al. 2018). Combining aerobic and resistance exercise is particularly recommended for the prevention and improved management of type 2 diabetes mellitus (T2DM) (Cosentino et al. 2020). High intensity interval training (HIIT) is another mode of exercise which results in improvements in cardiorespiratory fitness and cardiometabolic health in several populations including overweight and obese individuals (Batacan et al. 2017). Notably, a meta-analysis showed that HIIT can be a better exercise modality compared to moderate intensity continuous exercise (MICON) for the improvement of vascular function and the related biomarkers in clinical populations (Ramos et al. 2015).

Previous systematic reviews (Ribeiro et al. 2013; Palmefors et al. 2014) and meta-analyses (Pearson and Smart 2017; Cavalcante et al. 2019) have investigated the impact of long-term exercise on the number of circulating EPCs in CVD patients only. Nevertheless, little attention was placed on the exercise prescription configurations that induce optimum EPC mobilisation. It is also worth noting that the key pro-angiogenic factors that mediate exercise-induced EPC mobilisation have been systematically extracted and discussed alongside EPCs only in systematic reviews conducted until 2012 (Ribeiro et al. 2013; Palmefors et al. 2014). Finally, the acute and chronic effects of different exercise modalities in populations with impaired metabolic health (i.e., diabetes mellitus and metabolic syndrome) have not been systematically reviewed previously.

Therefore, the primary objective of this review is to systematically investigate the acute and chronic effects of different exercise modalities on circulating EPCs in patients with CVD and metabolic disease. A secondary objective is to investigate the available evidence with regards to the interplay between endothelial function, angiogenic factors and EPCs in individuals with CVD and metabolic diseases across the lifespan.

## Methods

This systematic review was conducted according to the PRISMA (Preferred Reporting Items for Systematic Reviews and Meta-analyses) statement (Moher et al. 2009) and was prospectively registered in the PROSPERO database (CRD42017084552).

### Literature search

A systematic literature search was conducted from 1996 until May 2018 using selected databases (MEDLINE, Cochrane Library (CENTRAL), SPORTdiscus, CINAHL, PsycINFO and SCOPUS). An update of the literature published between May 2018 to February 2020 and February 2020 to April 2021 was conducted using the MEDLINE database. Reference lists of existing reviews and eligible articles were used to assist the identification of eligible articles. Keyword searches for “population”, “exercise” and “endothelial progenitor cells” were performed using Boolean operators and wild cards where appropriate [Details of the strategy can be found in Supplementary Table 1 (S1)] No language restrictions were made. Findings reported here relate to the clinical populations of the registered review in the PROSPERO database however the search strategy included terms for both clinical and healthy populations.

### Study selection

The present review included randomised (RCTs) and non-randomised controlled trials (non-RCTs), prospective cohort studies, controlled before—after studies and without control before—after studies in human interventions that investigated the acute and chronic effects of different modes of exercise on circulating EPCs.

Study eligibility was determined by the following key inclusion criteria: (1) inclusion of individuals with CVD, metabolic disease, (2) implementation of structured exercise training programmes or acute exercise training bouts and (3) studies that implemented flow cytometry as main method to enumerate circulating EPCs. The criteria for the EPC phenotype was to include at least one marker of immaturity/stemness (i.e., CD34) and at least one marker that represent endothelial lineage (i.e., KDR) (Fadini et al. 2008a).

Studies not eligible for inclusion: animal studies, human studies where the participants were under 18 years old or pregnant women or individuals who were dieting.

Citations along with abstracts were transferred to EndNote version X9 and duplicates were removed. Two reviewers (PF and MS) independently screened and assessed the title and abstract of all eligible studies.

### Data extraction and quality assessment

After reviewing the full paper of all eligible studies, the data were extracted using a standardised extraction sheet in Microsoft excel (Office 365 Plus) by three independent reviewers (PF, CT and MS) and included: (1) Study information (Author, year); (2) Study population (clinical condition, age, sex, fitness status); (3) Exercise intervention (Acute; defined as a single bout of physical activity (Sellami et al. 2018)/Chronic; defined as repeated number of bouts of physical activity during short or long-term period of time (Sellami et al. 2018); (4) Exercise protocol (type of exercise, intensity, duration); (5) Primary outcomes (EPC phenotype, unit of measure, blood sampling time); (6) Secondary outcomes [cytokines, growth factors, chemokines, FMD, maximal oxygen uptake ($V{\text{O}}_{2\max }$)]. After data extraction, a meeting was held by the three reviewers to cross-check the extracted data. Any disagreements were resolved by discussion. Data not provided in the text or tables were extracted from figures using a semi-automated graph digitizer software (WebPlotDigitizer).

Study quality was assessed by PF and TI, using different critical appraisal tools appropriate for each study design. For the RCTs and non-RCTs, the TESTEX (Tool for the assEssment of Study qualiTy and reporting in Exercise) appraisal tool was used, which is a 15-point scale designed specifically for exercise intervention trials (Smart et al. 2015). For prospective cohort studies, controlled before—after studies and before—after studies the respective quality assessment tools from the National Heart, Lung and Blood institute were used (NHLBI 2021).

### Evidence synthesis

A narrative synthesis was undertaken, constructing evidence tables of key study characteristics along with an accompanying narrative synthesis across studies, in accordance with the Synthesis without meta-analysis in systematic reviews: reporting guideline (Campbell et al. 2020). The extracted data presented in two broad categories and in table format: trials that investigated the acute effects of exercise on EPCs and trials that investigated the chronic effects of exercise on EPCs. Tables then were thematically divided based on the exercise modality utilised and the clinical condition. Tables regarding the number of blood collection points and fasting/non-fasting status were arranged in alphabetical order. Quality assessment results were arranged by decreasing order based on the quality score.

The graphical abstract and Fig. 2 were created with BioRender.com. Due to the diversity in study designs, methodological analysis on EPCs (e.g. different phenotypes, different units of measure, different processing and time of blood collection) a meta-analysis was not conducted. Instead, extracted result data along with the P values from the papers were reported to assist the narrative synthesis and interpretation.

## Results

**Fig. 1:**
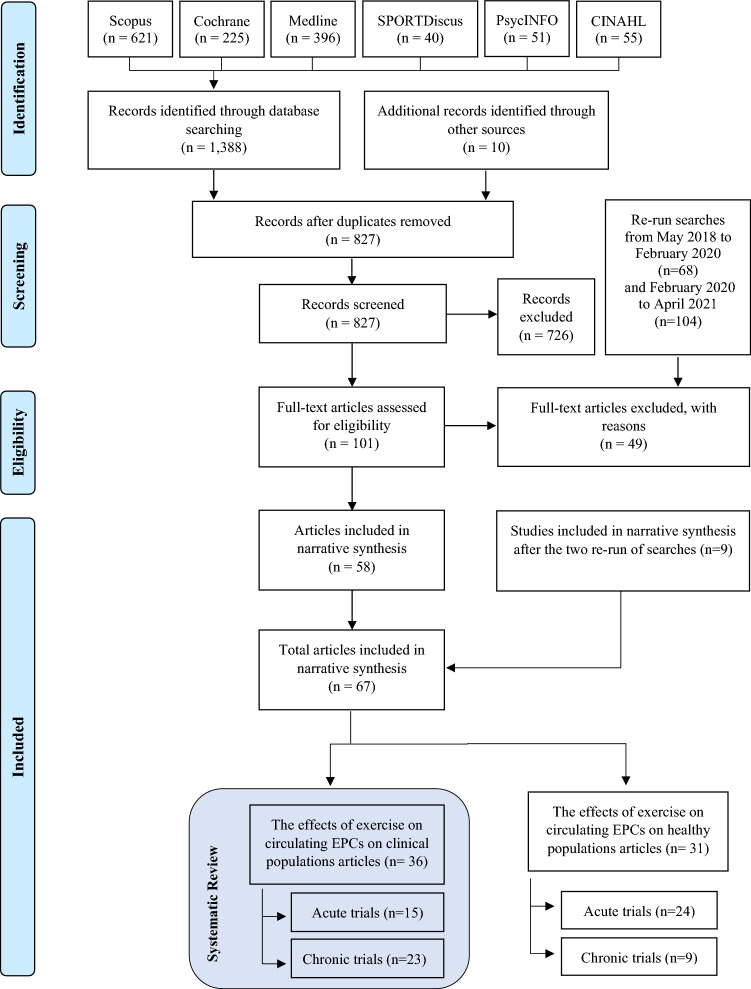
PRISMA flow diagram illustrating the searching strategy and selection of the articles used in this systematic review

The initial electronic search identified 1388 articles and the manual search from previous reviews another 10, in total 1398 articles. After exclusion of duplicates, 827 articles were reviewed based on title and abstract and after the first sifting 101 potentially eligible articles remained for full-text screening. This resulted in 58 articles that met the inclusion criteria. Following the two updated searches, nine additional eligible articles were identified, increasing the total number of articles to 67. A full list of the 49 articles, accompanied by reason, excluded for this review can be found as Supplementary Table 2 (S2). Figure 1 shows the PRISMA flow diagram of the screening process. Thirsty-six of the articles that met the inclusion criteria were included in this systematic review which focuses on populations with CVD and metabolic abnormalities. The remainder will form the basis for a systematic review that addressing the effects on healthy individuals across the lifespan.

### Overview of the study characteristics

The 36 articles yielded 38 trials as one of the publications included three RCT trials (Sandri et al. 2005). The total number of participants was 1731. Thirty-three trials (1588 participants) reported sex distribution with 1247 males (78.5%) and 341 females (21.5%) respectively. Five trials did not report sex distribution (Sandri et al. 2005; Adams et al. 2004; Shaffer et al. 2006).

### Acute clinical trial characteristics and intervention details

Fifteen clinical trials examined the acute effects to exercise (Adams et al. 2004; Shaffer et al. 2006; Van Craenenbroeck et al. 2009; Van Craenenbroeck et al. 2010a; Sandri et al. 2011; Van Craenenbroeck et al. 2011; Rummens et al. 2012; Scalone et al. 2013; Kazmierski et al. 2015; Rocha et al. 2015; West et al. 2015; Lutz et al. 2016; Waclawovsky et al. 2016; Gevaert et al. 2019; Kourek et al. 2020b) (Table 1). Thirteen trials included independent groups before and after (Adams et al. 2004; Shaffer et al. 2006; Van Craenenbroeck et al. 2009; Van Craenenbroeck et al. 2010a; Van Craenenbroeck et al. 2011; Rummens et al. 2012; Scalone et al. 2013; Kazmierski et al. 2015; Rocha et al. 2015; West et al. 2015; Lutz et al. 2016; Gevaert et al. 2019; Kourek et al. 2020b), one was a single arm trial (Sandri et al. 2011) and another one a randomised cross over trial (Waclawovsky et al. 2016). Across the 15 included trials (649 participants), 13 reported percentages of male and female distribution (Van Craenenbroeck et al. 2009; Van Craenenbroeck et al. 2010a; Sandri et al. 2011; Van Craenenbroeck et al. 2011; Rummens et al. 2012; Scalone et al. 2013; Kazmierski et al. 2015; Rocha et al. 2015; West et al. 2015; Lutz et al. 2016; Waclawovsky et al. 2016; Gevaert et al. 2019; Kourek et al. 2020b). Across these 13 trials (573 participants), 435 were males (75.9%) and 138 were females (24.1%). A healthy exercise control group was included in 12 out of the 15 acute clinical trials (Adams et al. 2004; Shaffer et al. 2006; Van Craenenbroeck et al. 2010a; Van Craenenbroeck et al. 2011; Rummens et al. 2012; Scalone et al. 2013; Kazmierski et al. 2015; Rocha et al. 2015; West et al. 2015; Lutz et al. 2016; Waclawovsky et al. 2016; Gevaert et al. 2019). The total healthy control were 199 participants (33.7%), whereas the total clinical population was 450 participants (69.3%). The age of the clinical population ranged from 27 to 74 and of the healthy controls from 27 to 73 years old. The clinical conditions for the acute trials included: four with CHF with reduced ejection fraction (HFrEF) and mid-ranged ejection fraction (HFmrEF) (Van Craenenbroeck et al. 2009, 2010a, 2011; Kourek et al. 2020b), one CHF with preserved ejection fraction (HFpEF) (Gevaert et al. 2019), four with coronary artery disease (CAD) and microvascular angina (Adams et al. 2004; Rummens et al. 2012; Scalone et al. 2013; Kazmierski et al. 2015), two with peripheral arterial disease (PAD) (Shaffer et al. 2006; Sandri et al. 2011), two with T1DM (West et al. 2015; Waclawovsky et al. 2016), one with T2DM and impaired glucose tolerance (Lutz et al. 2016) and one with early metabolic syndrome (MetS) (Rocha et al. 2015).

**Table 1:** Summary of trials examining the acute effects of different exercise modalities on EPCs in clinical populations EPCs

Study	Study design	Participant characteristics	Exercise Prescription	EPC phenotype identified by flow cytometry and units in brackets	Results on circulating EPCs and other major findings
Trials that employed a maximal exercise test on a cycle ergometer					
Adams et al. (2004)	Independent groups, before and after	EX group 1: *n* = 16 ischaemic CAD patients, gender not reported, 65 ± 1.4 yrs, Watt Max: 128 ± 6.4 W. EX group 2: *n* = 12 non-ischaemic CAD patients, gender not reported, 60 ± 2.1 yrs, Watt Max: 180 ± 15.2 W. EX group 3: *n* = 11 healthy participants, gender not reported, 59 ± 4.4 yrs, training status: Watt Max: 207 ± 19.9 W	Modality: Symptom limited EX test on cycle ergometer; Duration: EX group 1: 8.18 ± 6.2 min. EX group 2: 9.43 ± 8.4 min. EX group 3: 12.40 ± 1.15 min; Intensity: Maximal	CD34^+^/KDR^+^ (Cells/mL)	Significant ↑(164%) in EPCs up to 48 h( 76%) in EX group 1: Pre 73.9 ± 7.6 vs 24 h Post 194.9 ± 37.6, *P* = 0.001. Pre 73.9 ± 7.6 vs 48 h Post 130.0 ± 28.8, *P* = 0.001. EX group 2: Pre 77.4 ± 13.1 vs 24 h Post 69.0 ± 6.2, *P* > 0.05. Pre 77.4 ± 13.1 vs 48 h Post 63.8 ± 5.0, *P* > 0.05. EX group 3: Pre 97.6 ± 18.7 vs 24 h Post 107.9 ± 25.1, *P* > 0.05. Pre 97.6 ± 18.7 vs 48 h Post 108.3 ± 31.5, *P* > 0.05. ↑VEGF (EX group 1); ↔ GM-CSF, ↔ b-FGF, and ↔ TNF-a (all groups)
Gevaert et al. (2019)	Independent groups, before and after	EX group 1: *n* = 26 CHF patients with preserved ejection fraction ≥ 50%, 38% males, 74 ± 7 yrs, $V{\text{O}}_{{2{\text{peak}}}}$: 17.5 (13.6–19.1) mL kg^−1^ min^−1^. EX group 2: *n* = 26 sedentary healthy participants, 38%males, 73 ± 6 yrs, $V{\text{O}}_{{2{\text{peak}}}}$: 23.3 (21.6–29.0) mL kg^−1^ min^−1^	Modality: CPET on cycle ergometer; Duration: not reported; Intensity: Maximal	CD34^+^/KDR^+^/CD45^dim^ (Cells/10^6^ MNCs)	No change after exercise in both groups. EX group 1: Pre 41.2 (24.7; 65.3) vs Post 30.6 (15.3; 73.5), *P* > 0.05. EX group 2: Pre 62.9 (40.0; 86.5) vs Post 75.3 (50.6; 95.9), *P* > 0.05
Kourek et al. (2020b)	Independent groups, before and after	EX group 1: *n* = 25 patients with CHF, NYHA II-III, and LVEF of 32 ± 9%, 84%males, 57 ± 10 yrs, $V{\text{O}}_{{2{\text{peak}}}}$: 14.5 ± 2.5 mL kg^−1^ min^−1^. EX group 2: *n* = 24 patients with CHF, NYHA II-III, and LVEF of 32 ± 8%, 83%males, 55 ± 9 yrs, $V{\text{O}}_{{2{\text{peak}}}}$: 21.8 ± 2.4 mL kg^−1^ min^−1^	Modality: CPET on cycle ergometer; Duration: not reported; Intensity Maximal	CD34^+^/CD45^−^/CD133^+^/KDR^+^ and CD34^+^/CD133^+^/KDR^+^ and CD33^+^/CD45^−^/CD133^−^KDR^+^ (Cells/10^6^ enucleated cells)	Significant elevation in EPCs in both groups apart from CD34^+^/CD133^+^/KDR^+^ EPCs in EX group 1. EX group 1: CD34^+^/CD45^−^/CD133^+^/KDR^+^: Pre 1 (1–4) vs Post 5 (3–8), *P* < 0.001.- CD34^+^/CD133^+^/KDR^+^: Pre 13 (9–17) vs Post 13 (9–26), *P* > 0.05 - CD33^+^/CD45^−^/CD133^−^KDR^+^: Pre 2 (1–2) vs Post 3 (2–5), *P* < 0.001. EX group 2: CD34^+^/CD45^−^/CD133^+^/KDR^+^: Pre 2 (1–3) vs Post 6 (3–9), *P* < 0.001. CD34^+^/CD133^+^/KDR^+^: Pre 10 (7–18) vs Post 14 (10–19), *P* < 0.01. CD33^+^/CD45^−^/CD133^−^KDR^+^: Pre 1 (1–2) vs Post 4 (2–6), *P* < 0.001 EPC mobilisation not different when groups divided based on VE/VCO_2_ slope and LVEF. Relationship between exercise-induced changes in EPCs with $V{\text{O}}_{{2{\text{peak}}}}$ (*r* = 0.341, *P* = 0.017) (pooled group data).
Rummens et al. (2012)	Independent groups, before and after	EX group 1: *n* = 60 CAD patients after revascularisation (*n* = 30 CABG, *n* = 30 PCI), 100%males, 61 ± 1 yrs, $V{\text{O}}_{{2{\text{peak}}}}$: 1439 ± 54 ml min^1^. EX group 2: *n* = 25 healthy participants, 100%males, 55 ± 1 yrs, $V{\text{O}}_{{2{\text{peak}}}}$: 2936 ± 235 ml min^−1^	Modality: CPET on cycle ergometer; Duration: Not reported; Intensity: Maximal	CD34^+^/KDR^+^ And CD34^+^/CD133^+^/KDR^+^ and CD34^+^/CD133^−^/KDR^+^ (Cells/mL)	Significant elevation on CD34^+^/KDR^+^ and CD34^+^/CD133^−^/KDR^+^ EPCs in both groups. The increase in CD34^+^/CD133^−^/KDR^+^ EPCs was significantly smaller in CAD group compared to healthy group. EX group 1: CD34^+^/KDR^+^: Pre 122.7 ± 42.2 vs Post 143.8 ± 14.1, *P* < 0.05. -CD34^+^/CD133^+^/KDR^+^: Pre 62.2 ± 32.61 vs Post 67.6 ± 11.0, *P* > 0.05. CD34^+^/CD133^−^/KDR^+^: Pre 76.9 ± 23.1 vs Post 94.8 ± 9.6, *P* < 0.05. EX group 2: CD34^+^/KDR^+^: Pre 205.8 ± 79.6 vs Post 237.5 ± 25.9, *P* < 0.05. CD34^+^/CD133^+^/KDR^+^: Pre 61.9 ± 34.6 vs Post 47.4 ± 14.37, *P* > 0.05. CD34^+^/CD133^−^/KDR^+^: Pre 147.9 ± 48.9 vs Post 193.4 ± 16.2, *P* < 0.05
Van Craenenbroeck et al. (2009)	Independent groups, before and after	EX group 1: *n* = 10 patients with CHF type D personality, NYHA II-III, LVEF of 26.5 ± 2.1%, 100% males, 56.3 ± 3.0 yrs, $V{\text{O}}_{{2{\text{peak}}}}$: 23.7 ± 2.4 mL kg^1^ min^1^. EX group 2: *n* = 25 patients with CHF, NYHA I-III, LVEF of 27.6 ± 1.9%, 100%males, 63.4 ± 2.6 yrs, $V{\text{O}}_{{2{\text{peak}}}}$: 20.5 ± 2.0 mL kg^1^ min^1^	Modality: CPET on cycle ergometer; Duration: 8-10 min; Intensity: Maximal	CD34^+^/KDR^+^ (% lymphocytes)	No significant alteration of EPCs in any of the two groups. EX group 1: Pre 0.084 ± 0.055 vs Post 0.183 ± 0.029, *P* > 0.05. EX group 2: Pre 0.14 ± 0.03 vs Post 0.17 ± 0.03, *P* > 0.05
Van Craenenbroeck et al. (2010b)	Independent groups, before and after	EX group 1: *n* = 19 patients with severe CHF, NYHA II-III, LVEF of 22.8 ± 1.8%, 79%males, 63.0 ± 2.6 yrs, $V{\text{O}}_{{2{\text{peak}}}}$: 17.1 ± 1.3 mL kg^1^ min^1^. EX group 2: *n* = 22 patients with mild CHF, NYHA I-III, LVEF of 31.7 ± 0.9%, 83%males, 61.9 ± 2.5 yrs, $V{\text{O}}_{{2{\text{peak}}}}$: 21.8 ± 1.8 mL kg^1^ min^1^. EX group 3: *n* = 13 healthy participants, 69%males, 55.7 ± 1.6 yrs, $V{\text{O}}_{{2{\text{peak}}}}$: 45.0 ± 2.6 mL kg^1^ min^1^	Modality: CPET on cycle ergometer; Duration: 8-10 min; Intensity: Maximal	CD34^+^/KDR^+^/CD3^−^ (Cells/10^6^ events)	No significant alteration of EPCs in any of the three groups. EX group 1: Pre 106.1 ± 28.2 vs Post 109.4 ± 32, *P* = 0.875. EX group 2: Pre 116.4 ± 32.9 vs Post 219.6 ± 86.3, *P* = 0.198. EX group 3: Pre 186.1 ± 78.6 vs Post 179.2 ± 39.9, *P* = 0.933. ↑ SDF-1^α^ (in EX groups 2 and 3 only); ↔ VEGF (in all groups)
Van Craenenbroeck et al. (2011)	Independent groups, before and after	EX group 1: *n* = 7 patients with CHF, NYHA II-III, LVEF of 28.3 ± 2.9%, 71%males, 65.1 ± 3.5 yrs, $V{\text{O}}_{{2{\text{peak}}}}$: 15.5 ± 1.4 mL kg^−1^ min^−1^. EX group 2: *n* = 4 healthy participants, 100%males, 71.5 ± 3.0 yrs, $V{\text{O}}_{{2{\text{peak}}}}$: 26.4 ± 4.4 mL kg^−1^ min^−1^. EX group 3: *n* = 4 healthy participants, 100%males, 20.3 ± 0.9 yrs, $V{\text{O}}_{{2{\text{peak}}}}$: 47.7 ± 2.3 mL kg^−1^ min^−1^	Modality: CPET on cycle ergometer; Duration: not reported; Intensity: Maximal	CD34^+^/KDR^+^/CD3^−^ (Cells/10^6^ events)	Significant elevation in EPC numbers only in the old and young healthy groups after 24 h (*P* < 0.05). ↔ SDF-1α (in all groups)
Trials that employed a maximal exercise test on a treadmill					
Kazmierski et al. (2015)	Independent groups, before and after	EX group 1: *n* = 60 patients with premature CAD with NYHA I, 72%males, 43 (35–49) yrs, 9.9 METs. EX group 2: *n* = 33 healthy participants, 82%males, 42 (34–49) yrs, 10.1 METs	Modality: Standard Bruce test on treadmill; Duration: not reported; Intensity: Maximal	CD34^+^/CD133^+^/KDR^+^ (Cells/μL)	Significant ↑ in EPC numbers in both groups but delayed exercise-induced response in CAD group. EX group 1: Pre 2.1 (0–6.3) vs 15 min Post 2.1 (0.5–4.9), *P* > 0.05. Pre 2.1 (0–6.3) vs 60 min Post 3.2 (0.6–7.4), *P* = 0.00001. EX group 2: Pre 2.0 (0–5.1) vs 15 min Post 3.5(1.3–5.6), *P* = 0.00001. Pre 2.0 (0–5.1) vs 60 min Post 2.7 (1–4.8), *P* = 0.0002. Greater EPC increase after 60 min in patients with one-vessel disease
Sandri et al. (2011)	Single arm	EX group: *n* = 23 patients with stable PAOD, Fontaine IIb, 96%males, 61.4 ± 22.1 yrs, training status not reported	Modality: Symptom limited treadmill exercise test; Duration: not reported; Intensity: Maximal	CD34^+^/KDR^+^ and CD133^+^/KDR^+^ (Cells/mL)	Significant ↑ in CD34^+^/KDR^+^ and CD133^+^/KDR^+^ EPCs by 212% and 278% respectively after 24 h. CD34^+^/KDR^+^: Pre 82 ± 20 vs 24 h Post 256 ± 52, *P* = 0.035. CD133^+^/KDR^+^: Pre 65 ± 19 vs 24H Post 216 ± 53, *P* = 0.02.↑ VEGF; ↔ GM-GSF; ↔ b-FGF; ↔ TNF-a
Scalone et al. (2013)	Independent groups, before and after	EX group 1: *n* = 20 patients with microvascular angina, 50%males, 61 ± 10 yrs, training status not reported.EX group 2: *n* = 20 patients with stable angina CAD, 50%males, 63 ± 8 yrs, training status not reported. EX group 3: *n* = 20 healthy participants with ≥ 1CRVRF, 50%males, 59 ± 8 yrs, training status not reported	Modality: Standard treadmill Bruce test; Duration: EX group 1: 433 ± 121 s, EX group 2: 380 ± 104 s, EX group 3: 541 ± 135 s; Intensity: Maximal	CD34^+^/KDR^+^/CD45^−^ (Cells/10^5^ mononuclear cells)	No alteration in EPCs in any of the three groups. When groups combined, EPCs were increased significantly by 76.6% after 24 h. EX group 1: Pre 7.2 ± 6 vs 24 h Post 10 ± 15, *P* > 0.05. EX group 2: Pre 4.1 ± 5 vs 24 h Post 7.4 ± 15, *P* > 0.05. EX group 3: Pre 7.3 ± 7 vs 24 h Post 14.7 ± , *P* > 0.05. Whole group: Pre 6.2 ± 6.1 vs 24 h Post 10.7 ± 18, *P* = 0.027
Shaffer et al. (2006)	Independent groups, before and after	EX group 1: *n* = 15 PAD patients, gender not reported, 69 yrs, training status not reported. EX group 2: *n* = 13 healthy participants, gender not reported, 63 yrs, training status not reported. EX group 3: *n* = 9 healthy participants, gender not reported, 33 yrs, training status not reported	Modality: Gardner (EX 1 and 2) and Bruce treadmill protocols (EX 3); Duration: EX 1 and 2, 10 min max unless stopped due to symptoms. EX 3 15 min max; Intensity: Maximal	CD133^+^/KDR^+^ and CD34^+^/KDR^+^ and CD133^+^/CD34^+^/KDR ^+^ and CD133^+^/CD34^+^/KDR^+^/CD31 and CD34^+^/KDR^+^/CD146^+^/CD31^−^ (% live events)	Significant reduction by ~ 63% in CD133^+^/CD34^+^/KDR^+^/CD31^−^ EPCs in older healthy group (EX group 2), *P* < 0.05. No other significant change observed
Trials that included MICON exercise					
Lutz et al. (2016)	Independent groups, before and after	EX group 1: *n* = 17 patients with type 2 diabetes mellitus, 53%males, 60 ± 2 yrs, VO_max_: 22.1 ± 1.3 mL kg^−1^ min^−1^. EX group 2: *n* = 10 patients with impaired glucose tolerance, 50%males, 64 ± 2 yrs, VO_max_: 25.3 ± 1.8 mL kg^−1^ min^−1^. EX group 3: *n* = 18 participants with normal glucose tolerance, 39%males, 60 ± 2 yrs, VO_max_: 26.6 ± 1.3 mL kg^−1^ min^−1^	Modality: Aerobic exercise on treadmill; Duration: 30 min; Intensity: 60%$V{\text{O}}_{2\max }$	CD34^+^/KDR^+^ (Cells/10^6^ events)	Significant ↑ by 23% only in normal glucose tolerance group (EX group 3). EX group 1: Pre 24.41 ± 11.40 vs 30 min Post, *P* > 0.05. EX group 2: Pre 21.16 ± 15.11 vs 30 min Post, *P* > 0.05. EX group 3: Pre 62.32 ± 10.70 vs 30 min Post, *P* < 0.01.Relationship between exercise-induced changes in EPCs with $V{\text{O}}_{2\max }$ (*r* = 0.35, *P* = 0.02), groups as a whole
Rocha et al. (2015)	Independent groups, before and after	EX group 1: *n* = 15 participants with early MetS, 80%males, 37.0 ± 2.0 yrs, $V{\text{O}}_{{2{\text{peak}}}}$: 2.2 ± 0.3L min^−1^. EX group 2: *n* = 9 healthy participants, 67%males, 33.0 ± 3 yrs, $V{\text{O}}_{{2{\text{peak}}}}$: 2.3 ± 0.2L min^−1^	Modality: Aerobic exercise on cycle ergometer; Duration: 40 min; Intensity: 80% of ventilatory threshold	CD34^+^/KDR^+^ and CD133^+^/CD34^+^/VEGFR2^+^ (%)	Significant reduction in both EPC phenotypes after exercise in EX group 1 vs EX group 2 CD34^+^/KDR^+^: EX group 1 post 0.99 ± 0.31 vs EX group 2 post 2.33 ± 0.29, *P* = 0.02. CD133^+^/CD34^+^/VEGFR2^+^: EX group 1 post 0.20 ± 0.08 vs EX group 2 post 0.59 ± 0.18, *P* = 0.02. ↑ G-CSF (both groups); ↑ MMP-2 (healthy group); ↑ MMP-9 (early MetS group); ↔ VEGF (both groups); ↔ GM-CSF (both groups)
West et al. (2015)	Independent groups, before and after	EX group 1: *n* = 10 patients with type 1 diabetes mellitus, 100%males, 27 ± 2 yrs, $V{\text{O}}_{{2{\text{peak}}}}$: 51 ± 2.1 mL kg^−1^ min^−1^. EX group 2: *n* = 9 healthy participants, 100%males, 27 ± 1 yrs, $V{\text{O}}_{{2{\text{peak}}}}$: 50.7 ± 1.1 mL kg^−1^ min^−1^	Modality: Aerobic exercise on treadmill; Duration: 45 min Intensity: 70%$V{\text{O}}_{2\max }$	CD34^+^/KDR^+^/CD45^dim^ (Cells/100 Leucocytes)	Significant ↑ in EPCs 15 h post-exercise in healthy group. Significant % ↑ in EPCs 15 h post-exercise in EX group 2 compared to EX group 1 (139% vs 27%, *P* = 0.01). EX group 1: Pre 0.0012 ± 0.0014 vs 60 min Post 0.0013 ± 0.0013, *P* > 0.05. Pre 0.0012 ± 0.0014 vs 15 h Post 0.0020 ± 0.0015, *P* > 0.05. EX group 2: Pre 0.0009 ± 0.0028 vs 60 min Post 0.0013 ± 0.0034, *P* < 0.05. Pre 0.0009 ± 0.0028 vs 15 h Post 0.0024 ± 0.0014, *P* < 0.05. ↔ TNF-α (both groups). Inverse relationship between ∆ change in EPCs from rest to 15 h Post-exercise with HbA1C (*r* = – 0.65, *P* = 0.021) and TNF-α (*r* = – 0.766, *P* = 0.005)
Waclawovsky et al. (2016)	Randomised cross over	EX group 1: *n* = 14 patients with type 1 diabetes mellitus, 100%males, 30.3 ± 1.6 yrs, $V{\text{O}}_{{2{\text{peak}}}}$: 37.1 ± 1.4 mL kg^−1^ min^−1^. EX group 2: *n* = 5 healthy participants, 100%males, 26.8 ± 2.3 yrs, $V{\text{O}}_{{2{\text{peak}}}}$: 42.3 ± 2.7 mL kg^−1^ min^−1^	Modality 1: 30 min cycling (60%$V{\text{O}}_{{2{\text{peak}}}}$) Modality 2: 40 min lower limb resistance exercise (4 exercises × 4sets × 12reps at 60%1RM and 90 s rest between sets)	CD34^+^/KDR^+^/CD45^dim^ (Log scale)	Significant ↑ in EPCs after resistance exercise in healthy group (EX group 2), *P* = 0.004. Significant reduction in EPCs after aerobic exercise in healthy group (EX group2), *P* = 0.017. No changes on Type 2 diabetes group (EX group 1), *P* > 0.05

*b-GFG* basic fibroblast growth factor, *CABG* coronary bypass artery grafting, *CAD* coronary artery disease, *CHF* chronic heart failure, *CPET* cardiopulmonary exercise test, *CRVRF* cardiovascular risk factors, *EPCs* endothelial progenitor cells, *EX* exercise, *G-CSF *granulocyte colony stimulating factor, *GM-CSF *granulocyte macrophage colony stimulating factor, *HbA1C* glycated haemoglobin, *LVEF* left ventricular ejection fraction, *ml* millilitres, *METS* metabolic equivalents, *MetS* metabolic syndrome, *min* minutes, *MICON* moderate intensity continuous exercise, *MMP -2/9 *matrix metalloproteinase 2/9, *MNCs* mononuclear cells, *NYHA* New York Heart Association classification, *PAOD* peripheral arterial occlusive disease, *PCI *percutaneous coronary intervention, *SDF-1α *stromal-cell derived factor 1 alpha, *TNF-α *tumour necrosis factor 1 alpha, *VEG*F vascular endothelial growth factor, *VE/VCO*_*2*_* slope *minute ventilation to carbon dioxide production relationship, *VO*_*2peak/max*_ peak/max oxygen uptake, *W* watts, *yrs* years, *1RM* one repetition maximum strength, ↑ indicates significant increase, ↔ indicates no significant change

The most common exercise mode for studying the acute effects was a cardiopulmonary exercise test (CPET) on a cycle ergometer (Adams et al. 2004; Van Craenenbroeck et al. 2009, 2010a, 2011; Rummens et al. 2012; Gevaert et al. 2019; Kourek et al. 2020b). Three trials utilised a standard Bruce test and a Gardner–Skinner stress test on a treadmill (Shaffer et al. 2006; Scalone et al. 2013; Kazmierski et al. 2015), and one trial utilised a symptom-limited maximal test on a treadmill (Sandri et al. 2011). With regards to MICON exercise on a cycle ergometer, intensity ranged from 60 to 70% $V{\text{O}}_{2\max }$ and duration between 30 and 45 min in two trials (West et al. 2015; Lutz et al. 2016), and one trial prescribed 40 min of cycling at 80% of the ventilatory threshold (Rocha et al. 2015). Finally, one trial compared a MICON protocol with a lower limb resistance exercise protocol matched in total duration as the MICON (Waclawovsky et al. 2016).

In total, eleven different phenotypes were used to identify circulating EPCs in the acute trials. The most common antibody combination was CD34^+^/KDR^+^ (Adams et al. 2004; Shaffer et al. 2006; Van Craenenbroeck et al. 2009; Sandri et al. 2011; Rummens et al. 2012; Rocha et al. 2015; Lutz et al. 2016). The remaining trials used: CD34^+^/CD133^+^/KDR^+^ (5 trials) (Shaffer et al. 2006; Rummens et al. 2012; Kazmierski et al. 2015; Rocha et al. 2015; Kourek et al. 2020b), CD34^+^/KDR^+^/CD45^dim^ (3 trials) (Gevaert et al. 2019; Waclawovsky et al. 2016; West et al. 2015), CD34^+^/KDR^+^/CD3^−^ (2 trials) (Van Craenenbroeck et al. 2010a, 2011), CD133^+^/KDR^+^ (2 trials) (Shaffer et al. 2006; Sandri et al. 2011), CD34^+^/CD133^−^KDR^+^ (1 trial) (Rummens et al. 2012) and CD34^+^/KDR^+^/CD45^−^ (Scalone et al. 2013) (1 trial). Finally, two trials used two versions of four antibody combinations including CD133^+^/CD34^+^/KDR^+^/CD31^−^ or CD34^+^/KDR^+^/CD146^+^/CD31^−^ (Shaffer et al. 2006) and CD34^+^/CD45^−^/CD133^+^/KDR^+^ or CD34^+^/CD45^−^/CD133^−^/KDR^+^ (Kourek et al. 2020b) respectively. Large variability was observed in terms of the units used to express EPCs. The most common units were the absolute number of cells (cells/mL or cells/μL) (Adams et al. 2004; Sandri et al. 2011; Rummens et al. 2012; Kazmierski et al. 2015) and cells per 10^6^ events (Van Craenenbroeck et al. 2010a, 2011; Lutz et al. 2016). Several other units of EPCs identified, such as percentage lymphocytes (Van Craenenbroeck et al. 2009), cells per 10^6^ mononuclear cells (Gevaert et al. 2019), cells per 10^5^ mononuclear cells (Scalone et al. 2013), percentage of live events (Shaffer et al. 2006), cells per 100 leucocytes (West et al. 2015), percentage cells in the CD34^+^ gate (Rocha et al. 2015) and cells per 10^6^ enucleated cells (Kourek et al. 2020b). One trial presented EPCs in logarithmic scale (Waclawovsky et al. 2016). Lastly, as a secondary outcome four trials measured the number and/or function of cultured myeloid angiogenic cells (MACs) defined as double positive for acetylated Low-Density Lipoprotein labelled with 1,1′-dioctadecyl-3,3,3′,3′-tetramethyl-indocarbocyanine perchlorate and lectin (Di-acLDL ^+^ /lectin ^+^ cells) (Adams et al. 2004; Van Craenenbroeck et al. 2009, 2010a; Sandri et al. 2011) [Supplementary Table 3 (S3)].

Ten out of the 15 trials (Table 2) predominantly included two blood collection time points for circulating EPCs with the post-exercise time point varying between immediately post-exercise and 24 h post-exercise (Shaffer et al. 2006; Van Craenenbroeck et al. 2009; Van Craenenbroeck et al. 2010a; Rummens et al. 2012; Scalone et al. 2013; Rocha et al. 2015; Lutz et al. 2016; Waclawovsky et al. 2016; Gevaert et al. 2019; Kourek et al. 2020b). The most common time point adopted in the majority of trials was 10 min post-exercise. Two trials included three time points (Kazmierski et al. 2015; West et al. 2015). Three trials investigated the kinetics of circulating EPCs after exercise, whereas one included seven time points up to 48 h post-exercise (Adams et al. 2004), and two had 10 time points up to 48 h and 72 h post-exercise respectively (Sandri et al. 2011; Van Craenenbroeck et al. 2011). Four trials reported fasting (Shaffer et al. 2006; Rocha et al. 2015; Lutz et al. 2016; Waclawovsky et al. 2016), and three trials reported non-fasting blood samples (Van Craenenbroeck et al. 2010a, 2011; Rummens et al. 2012). One trial reported pre-exercise non-fasting blood sampling and the ~ 15 h post-exercise fasting blood sample (West et al. 2015). Finally, seven trials did not report any details (Adams et al. 2004; Van Craenenbroeck et al. 2009; Sandri et al. 2011; Scalone et al. 2013; Kazmierski et al. 2015; Gevaert et al. 2019; Kourek et al. 2020b) (Table 2).

**Table 2:** Summary of blood collection points and in fasting and non-fasting state in acute trials

Study	Number of blood samples	Time point of blood collection	Fasting/non-fasting status
Adams et al. (2004)	7	Pre-Exercise, 2 h, 4 h, 6 h, 8 h, 24 h, and 48 h Post-Exercise	Not reported
Gevaert et al. (2019)	2	Pre-Exercise and 10 min Post-Exercise	Not reported
Kazmierski et al. (2015)	3	Pre-Exercise, 15 min, 60 min Post-Exercise	Not reported
Kourek et al. (2020b)	2	Pre-Exercise and immediately Post-Exercise	Not reported
Lutz et al. (2016)	2	Pre-Exercise and 30 min Post-Exercise	Overnight fast (~ 12 h)
Rocha et al. (2015)	2	Pre-Exercise and 10 min Post-Exercise	12 h fast
Rummens et al. (2012)	2	Pre-Exercise and immediately Post-Exercise	Non fasting
Sandri et al. (2011)	10	Pre-Exercise, 2 h, 4 h, 6 h, 8 h, 10 h, 12 h, 24 h, 48 h, and 72 h Post-Exercise	Not reported
Scalone et al. (2013)	2	Pre-Exercise and 24 h Post-Exercise	Not reported
Shaffer et al. (2006)	2	Pre-Exercise and 10 min Post-Exercise	Overnight fast
Van Craenenbroeck et al. (2009)	2	Pre-Exercise and 10 min Post-Exercise	Not reported
Van Craenenbroeck et al. (2010a)	2	Pre-Exercise and 10 min Post-Exercise	Non fasting
Van Craenenbroeck et al. (2011)	10	Pre-Exercise, 10 min, 30 min, 1 h, 2 h, 4 h, 8 h, 12 h, 24 h, and 48 h Post-Exercise	Non fasting
Waclawovsky et al. (2016)	2	Pre-Exercise and 10 min Post-Exercise	2 h fast
West et al. (2015)	3	Pre-Exercise, 60 min, and 15 h Post-Exercise	No fasting Pre-Exercise. Fasting at ~ 15 h Post-Exercise sample

### Chronic clinical trial characteristics and intervention details

From the 38 clinical trials, 23 examined the chronic effects of exercise on circulating EPCs (Laufs et al. 2004; Sandri et al. 2005, 2016; Steiner et al. 2005; Paul et al. 2007; Sarto et al. 2007; Cesari et al. 2009, 2013; Erbs et al. 2010; Van Craenenbroeck et al. 2010b, 2015; Hansen et al. 2011; Schlager et al. 2011; Gatta et al. 2012; Luk et al. 2012; Eleuteri et al. 2013; Mezzani et al. 2013; Dopheide et al. 2016b; Gagliardi et al. 2016; Jo et al. 2020; Kourek et al. 2020a) (Table 3). Ten trials were RCTs (Sandri et al. 2005, 2016; Erbs et al. 2010; Schlager et al. 2011; Luk et al. 2012; Eleuteri et al. 2013; Mezzani et al. 2013; Gagliardi et al. 2016), six single arm before—after trials (Laufs et al. 2004; Paul et al. 2007; Sarto et al. 2007; Cesari et al. 2009, 2013; Gatta et al. 2012), four randomised trials (Hansen et al. 2011; Van Craenenbroeck et al. 2015; Jo et al. 2020; Kourek et al. 2020a) and three non-RCTs (Steiner et al. 2005; Van Craenenbroeck et al. 2010b; Dopheide et al. 2016b). Across the 23 included trials (1082 participants), 20 trials reported percentage of female and male distribution (Laufs et al. 2004; Steiner et al. 2005; Paul et al. 2007; Sarto et al. 2007; Cesari et al. 2009, 2013; Erbs et al. 2010; Van Craenenbroeck et al. 2010b, 2015; Hansen et al. 2011; Schlager et al. 2011; Gatta et al. 2012; Luk et al. 2012; Eleuteri et al. 2013; Mezzani et al. 2013; Dopheide et al. 2016b; Gagliardi et al. 2016; Sandri et al. 2016; Jo et al. 2020; Kourek et al. 2020a). Across these 20 trials (1015 participants), 812 (80.0%) were males and 203 (20%) were females. In chronic clinical interventions, age ranged from 44 to 88 years old. In chronic interventions, the clinical conditions included: HFrEF (8 trials) (Sarto et al. 2007; Erbs et al. 2010; Van Craenenbroeck et al. 2010b; Gatta et al. 2012; Eleuteri et al. 2013; Mezzani et al. 2013; Sandri et al. 2016; Kourek et al. 2020a), CAD (8 trials) (Laufs et al. 2004; Sandri et al. 2005; Steiner et al. 2005; Paul et al. 2007; Hansen et al. 2011; Luk et al. 2012; Van Craenenbroeck et al. 2015; Gagliardi et al. 2016), PAD (4 trials) (Sandri et al. 2005; Schlager et al. 2011; Dopheide et al. 2016b), cardiac patients (coronary artery bypass graft and valve replacement) (1 trial) (Cesari et al. 2009), acute coronary syndrome (ACS) (1 trial) (Cesari et al. 2013), and hypertensive MetS (1 trial) (Jo et al. 2020).

**Table 3:** Summary of trials examining the chronic effects of different exercise modalities on EPCs

Study	Study design	Participant characteristics	Exercise Prescription	EPC phenotype identified by flow cytometry and units in brackets		Results on circulating EPCs and other major findings
Trials that included MICON exercise						
Cesari et al. (2013)	Single arm	EX group: *n* = 112 acute coronary syndrome (74.1% STEMI, 25.9% NSTEMI) patients, 82%males, 58.2 ± 9.5 years, $V{\text{O}}_{{2{\text{peak}}}}$: 19.7 ± 5.7 ml kg^−1^ min^−1^	Length: 4 weeks; Frequency: 3×/week; Duration: 30 min; Modality: Aerobic; Intensity: 60–70%$V{\text{O}}_{{2{\text{peak}}}}$	CD34^+^/KDR^+^ and CD133^+^/KDR^+^ and CD34^+^/CD133^+^/KDR^+^ (Cells/10^6^ events)		Significant improvement on all EPC phenotypes. CD34^+^/KDR^+^: Pre 7 (0–30) vs Post 11 (0–37), *P* < 0.001. CD133^+^/KDR^+^: Pre 7 (0–27) vs Post (10 (0–33), *P* < 0.001. CD34^+^/CD133^+^/KDR^+^: Pre 7 (0–27) vs Post 10 (0–33), *P* < 0.001. ↑ $V{\text{O}}_{{2{\text{peak}}}}$_;_ ↓ hs-CRP; Significant relationship between ΔEPCs/Δ$V{\text{O}}_{{2{\text{peak}}}}$
Dopheide et al. (2016b)	Non-RCT	Supervised EX group: *n* = 20 patients with PAD, Rutherford 1–3, Fontaine II A/B, 75%males, 73.0 (60.8,76.0) years, Max. WD: median (IQR) (329; 619) m. Non- supervised EX group: *n* = 20 patients with PAD, Rutherford 1–3, Fontaine II A/B, 75%males, 66 (62.3; 72.8) years, Max. WD: median (IQR) 474 (432; 670) m	Length: 7.65 ± 1.62 months; Frequency: 3–5×/Week; Duration: 30–60 min; Modality: Aerobic; Intensity: Intensity which brings on claudication	CD34^+^/KDR^+^/CD45^dim^ (% positive cells)		Significant reduction in EPCs in both groups but with higher magnitude in supervised EX group. Supervised EX group: Pre 0.18 (0.14; 0.29) vs Post 0.06 (0.05; 0.09), *P* < 0.01. Non-Supervised EX group: Pre 0.20 (0. 13; 0.25) vs Post 0.11 (0.08; 0.17). *P* < 0.01. ↑ Max. WD (both groups); ↑ VEGF-A (supervised Ex group); ↓ CRP (supervised Ex group); relationship between individual changes in VEGF-A with changes in EPCs (*r* = – 0.477, *P* < 0.001)
Eleuteri et al. (2013)	RCT	Ex group: *n* = 11 CHF patients NYHA class II with LVEF ≤ 40%, 100% males, 66 ± 2 years, $V{\text{O}}_{{2{\text{peak}}}}$: 14.8 ± 0.7 mL kg^−1^ min^−1^. Control group: *n* = 10 CHF patients NYHA class II with LVEF ≤ 40%, 100%males, 63 ± 2 years, $V{\text{O}}_{{2{\text{peak}}}}$: 16.7 ± 0.4 mL kg^−1^ min^−1^	Length: 3 months; Frequency: 5 ×/week (home based); Duration: 30 min; Modality: Aerobic; Intensity: Cycling (60rev/min) at a power and heart rate corresponding to VAT	CD34^+^/KDR^+^/CD45^dim^ (%MNCs)		Significant ↑ in EPCs in the EX group only. EX group: Pre 0.013(0.005–0.032) vs Post 0.028(0.009–0.048), *P* = 0.025. Control group: Pre 0.012(0.005–0.052) vs Post 0.019(0.003–0.037), *P* = 0.44. ↑ $V{\text{O}}_{{2{\text{peak}}}}$ (EX group); ↑ FMD (EX group); ↑ Angiopoietin 2 (Ex group); ↔ Angiopoietin 1 (both groups); ↔ VEGF (both groups); ↔ SDF-1α (both groups); ↔ CRP (both groups); no relationship between EPCs with $V{\text{O}}_{{2{\text{peak}}}}$ and Ang-2
Erbs et al. (2010)	RCT	EX group: *n* = 18 CHF patients NYHA IIIb with LVEF ≤ 30%, 100%males, 60 ± 11 years, $V{\text{O}}_{2\max }$: 15.3 ± 3.3 ml kg^−1^ min^−1^. Control group: *n* = 19 CHF patients NYHA IIIb with LVEF ≤ 30%, 100%males, 62 ± 10 years, $V{\text{O}}_{2\max }$: 15.4 ± 3.8 ml kg^−1^ min^−1^	Length: 15 weeks (3 weeks supervised and 12 weeks at home) (results are for the pre/post 12 weeks); Frequency: Daily; Duration: During the 12 weeks 20-30 min; Modality: Aerobic; Intensity: Cycling at 60%$V{\text{O}}_{2\max }$	CD34^+^/KDR^+^ (Cells/mL)		Significant ↑ in EPC numbers by 83% versus control group. EX group: Pre 100.0 ± 127.0 vs Post 183 ± 156, *P* = 0.014 vs Control group: Pre 115.8 ± 89.7 vs Post 109.7 ± 93.3. ↑ $V{\text{O}}_{2\max }$ (EX group); ↑ FMD (EX group); ↑ VEGF (EX group);↑ SDF-1α (EX group); ↓ TNF-α (EX group)
Gatta et al. (2012)	Single arm	EX group: *n* = 14 patients with CHF, NYHA II with LVEF < 40%, 57%males, 72 ± 11 yearsy, 6MWT: 154 ± 27 m	Length: 3 weeks; Frequency: 2x/day for 6x/week; Duration: 30 min; Modality: Aerobic; Intensity: Cycling at 85%HRmax or at 75%HRmax for patients > 65 years	CD34^+^/KDR^+^ (% positive cells)		Significant ↑ in EPCs in the EX group. EX group: Pre 0.07 ± 0.01 vs Post 0.12 ± 0.01, *P* < 0.05. ↑ 6MWT; ↑ MMP-2/TIMP-1; ↑ MMP-9/TIMP-1; ↔ MMP-9; ↔ MMP-2; ↓ TNF-α; significant inverse correlation between EPCs with TNF-a: (*r* = – 0.778, *P* < 0.01)
Mezzani et al. (2013)	RCT	EX group: *n* = 15 CHF patients NYHA class II with LVEF ≤ 40%, 100% males, 65 ± 7 years, $V{\text{O}}_{{2{\text{peak}}}}$: 15.7 ± 4.2 ml kg^−1^ min^−1^. Control group: *n* = 15 CHF patients NYHA class II with LVEF ≤ 40%, 100% males, 63 ± 7 years, $V{\text{O}}_{{2{\text{peak}}}}$: 17.0 ± 1.6 ml kg^−1^ min^−1^	Length: 12 weeks; Frequency: 5×/week; Duration: 30 min; Modality: Aerobic; Intensity: Cycling at VT1(CPET repeated 6 weeks into training to adjust intensity)	CD34^+^/KDR^+^/CD45^dim^ (% MNCs)		Significant improvement in EPCs in the EX group only. EX group: Pre 0.013 ± 0.008 vs Post 0.037 ± 0.012, *P* < 0.05. Control group: Pre 0.025 ± 0.012 vs Post 0.019 ± 0.019, *P* > 0.05.↑ $V{\text{O}}_{{2{\text{peak}}}}$ (EX group); significant correlation between relative increase in EPCs and $V{\text{O}}_{{2{\text{peak}}}}$: (*r* = 0.64, *P* < 0.05)
Paul et al. (2007)	Single arm	EX group: *n* = 46 CAD patients with LVEF > 30%, 90% males, 44-80 years, training status not reported	Length: 12 weeks; Frequency: 3×/week; Duration: Progressed from ~ 25 min to 39 min; Modality: Aerobic; Intensity: not reported	CD133^+^/KDR^+^ (Cells/ml)		two-fold increase. Pre 35 ± 5 vs Post 63 ± 10, *P* < 0.05. ↑ Total exercise time; ↔ FMD; ↔ CRP
Sandri et al. (2005)	RCT	EX group: *n* = 9 patients with stable ischaemic PAOD, Fontaine IIb, gender: not reported, 56.0 ± 1.4 years, Max. WD: 152 ± 8 m. Control group: *n* = 9 patients with stable PAOD, Fontaine IIb , gender: not reported, 57.0 ± 2.1 years, Max.WD: 148 ± 12 m	Length: 4 weeks; Frequency: 6x/day for 5×/week; Duration: not reported; Modality: Aerobic; Intensity: Walking at 12% incline at 3.5 km.h^−1^ on treadmill	CD34^+^/KDR^+^ (Cells/mL)		5.2-fold significant ↑ in EPCs in the EX group only. EX group: Pre 90 ± 14 vs Post 468 ± 21, *P* < 0.05. Control group: Pre 85 ± 25 vs Post 72 ± 31, *P* > 0.05. ↑ Max. WD (EX group); ↑ VEGF (EX group); ↔ GM-CSF (both groups); ↔ TNF-α (both groups); relationship between VEGF and EPCs (*r* = 0.66, *P* < 0.05)
Sandri et al. (2005)	RCT	EX group: *n* = 9 patients with non-ischaemic prior PAOD, Fontaine IIb, gender: not reported, 63.0 ± 2.5 years, Max.WD: 335 ± 28 m. Control group: *n* = 9 patients with non-ischaemic prior PAOD, Fontaine IIb, gender: not reported, 62 ± 2 years, Max. WD: 367 ± 25 m	Length: 4 weeks; Frequency: 6x/day for 5×/week; Duration: Exercise at 75% of Max. WD; Modality: Aerobic; Intensity: Walking at 12% incline at 3.5 km.h^−1^ on treadmill	CD34^+^/KDR^+^ (Cells/mL)		No chance in EPC levels. EX group: Pre 74 ± 13 vs Post 91 ± 22, *P* > 0.05. Control group: Pre 94 ± 17 vs Post 89 ± 19, *P* > 0.05. ↑ Max. WD (EX group); ↔ VEGF (both groups); ↔ GM-CSF (both groups); ↔ TNF-α (both groups)
Sandri et al. (2005)	RCT	EX group: *n* = 15 stable CAD patients, gender: not reported, 62.4 ± 1.7 years, $V{\text{O}}_{{2{\text{peak}}}}$: 19.5 ± 2.5 ml kg^−1^ min^−1^. Control group: *n* = 16 stable CAD patients, gender: not reported, 59.0 ± 2.1 years, $V{\text{O}}_{{2{\text{peak}}}}$: 19.2 ± 2.3 ml kg^−1^ min^−1^	Length: 4 weeks; Frequency: 6x/day for 5×/week; Duration: 10 min; Modality: Aerobic; Intensity: Cycling at 70%HR_peak_	CD34^+^/KDR^+^ (Cells/mL)		No change in EPC levels. EX group: Pre 110 ± 16 vs Post 126 ± 15, *P* > 0.05. Control group: Pre 104 ± 17 vs 116 ± 14, *P* > 0.05. ↑ $V{\text{O}}_{2\max }$ (EX group); ↔ VEGF (both groups); ↔ GM-CSF (both groups); ↔ TNF-α (both groups)
Sarto et al. (2007)	Single arm	EX group: *n* = 22 CHF patients NYHA II-III with LVEF ≤ 40%, 73%males, 61.4 ± 1.6 years, $V{\text{O}}_{{2{\text{peak}}}}$:15.13 ± 0.79 ml kg^1^ min^−1^	Length: 8 weeks; Frequency: 3×/week; Modality: Aerobic; Duration: 55 min; Intensity: Cycling at 60% of HRR	CD34^+^/KDR^+^/CD31^+^(Cells/mL)		Significant ↑ by 152% in EPCs. Pre 88 ± 5.99 vs Post 221.36 ± 21.29, *P* < 0.05. ↑ $V{\text{O}}_{{2{\text{peak}}}}$; ↑ VEGF; ↑ SDF-1α
Schlager et al. (2011)	RCT	EX group: *n* = 20 symptomatic PAD, Rutherford categories I-III, 65%males, 69 ± 8 years; Max. WD: median (IQR) 101.5 (65.5; 154.5) m. Control group: *n* = 20 symptomatic PAD, Rutherford categories I-III , 55%males, 70 ± 11 years, Max. WD: median (IQR) 84.8 (50; 150) m	Length: 6 months; Frequency: 2x/week; Duration: 35 min increased by 5 min until 50 min achieved; Modality: Aerobic; Intensity: Intermittent walking at speed that elicited claudication symptoms within 3–5 min. Trained at this workload until moderate claudication and brief rest to alleviate symptoms	CD34^+^/KDR^+^/CD133^+^ (% MNCs)		Significant improvement in EPCs in the EX group only after 3 and 6 months. EX group: Pre 0.0016 (0; 0.0036) vs 3 months 0.0033 (0.0018; 0.0074), *P* < 0.05. Pre 0.0016 (0; 0.0036) vs Post 0.0030 (0.0017; 0.0063), *P* < 0.05. Control group: Pre 0.0017 (0.0013; 0.0032) vs 3 months 0.0015 (0.0006; 0.0018), *P* > 0.05. Pre 0.0017 (0.0013; 0.0032) vs Post 0.0017 (0; 0.0033), *P* > 0.05. ↑ Max. WD (EX group); ↓ plasma ADMA (EX group only); ↔ serum VEGF (both groups); ↔ serum SDF-1α (both groups)
Steiner et al. (2005)	Non-RCT	EX group: *n* = 20 asymptomatic CAD and/or CVRFs patients, 80% males, 52 ± 10 years, 2 km test run: 20 ± 0.7 min. Control group: *n* = 20 CAD and/or CVRFs patients, 80%males, 52 ± 10 years, training status: not reported	Length: 12 weeks; Frequency: 3×/week individual plus 2x/week supervised; Duration: 30-60 min; Modality: Aerobic; Intensity: In line with current guidelines	CD34^+^/KDR^+^/CD133^+^ (% positive cells)		Significant ↑ in EPC numbers by 2.9 ± 0.4 fold in EX group only. EX group: Pre 0.0030 ± 0.0005 vs Post 0.0076 ± 0.0008, *P* < 0.001. Control group: Pre 0.0032 ± 0.0005 vs Post 0.0033 ± 0.0006, *P* > 0.05 ↑ 2 km exercise performance; ↑ NOx production (EX group); ↑ VEGF (EX group); ↔ FMD. Relationship between ΔNOx with ΔEPCs: *r* = 0.83, *P* < 0.01 and Δ%FMD with ΔEPCs: (*r* = 0.81, *P* < 0.01)
Trials that combined aerobic with resistance exercise						
Hansen et al. (2011)	Randomised trial	EX group 1: *n* = 25 revascularized CAD patients, 92% males, 58.9 ± 7.2 years, $V{\text{O}}_{{2{\text{peak}}}}$: 1836 ± 480 mL min^−1^. EX group 2: *n* = 22 revascularized CAD patients, 95.5% males, 60.4 ± 8.9 years $V{\text{O}}_{{2{\text{peak}}}}$: 1719 mL min^−1^	Length: 6 weeks; Frequency: 3×/week; Duration: 40 min; Modality: EX group 1: aerobic (cycling, walking, arm cranking). EX group 2: aerobic + lower limb resistance (leg extension, leg press); Intensity: EX group 1:65%$V{\text{O}}_{{2{\text{peak}}}}$_,_ EX group 2: 65% $V{\text{O}}_{{2{\text{peak}}}}$ and 2 × 12 reps at 65%1RM	CD34^+^/KDR^+^ and CD34^+^CD133^+^/KDR^+^ (Cells/mL)		No change in EPCs in any of the two exercise groups. CD34^+^/KDR^+^: EX group 1: Pre 94 ± 169 vs Post 129 ± 302, *P* > 0.05. EX group 2: Pre 37 ± 78 vs Post 33 ± 65, *P* > 0.05. CD34^+^CD133^+^/KDR^+^: EX group 1: Pre 19 ± 44 vs Post 19 ± 46, *P* > 0.05. EX group 2: Pre 5 ± 7 vs Post 5 ± 7, *P* > 0.05. ↑ $V{\text{O}}_{{2{\text{peak}}}}$ (both groups); ↔ CRP (both groups)
Laufs et al. (2004)	Single arm	EX group: *n* = 19 stable CAD patients, 47%males, 70.1 ± 7.7 years, $V{\text{O}}_{{2{\text{peak}}}}$: 9.58 ± 4.0 ml kg^−1^ min^−1^, 6MWT: 357 ± 98 m	Length: 28 days; Frequency: Weekly training (lower limb strength training 2–3 times, cycling 3 times, 6MWT as training twice); Modality: Aerobic and resistance; Intensity: Moderate strength training, cycling at HR corresponding to 60–80% $V{\text{O}}_{{2{\text{peak}}}}$	CD34^+^/KDR^+^ (Cells/10^5^ events)		Significant ↑ in EPCs by 78 ± 34%. Pre 23.13 ± 5.87 vs Post 40.73 ± 12.22, *P* < 0.05. ↑ $V{\text{O}}_{{2{\text{peak}}}}$_;_ ↑ 6MWT
Luk et al. (2012)	RCT	EX group: *n* = 32 CAD patients with > 50% stenosis in at least one coronary artery, 75%males, 67.7 ± 9.0 years, 8.52 ± 2.97 METs. Control group: *n* = 32 CAD patients with > 50% stenosis in at least one coronary artery, 75%males, 66.6 ± 7.9 years, 7.82 ± 2.20 METs	Length: 8 weeks; Frequency: 3×/week; Duration: 50 min; Modality: Aerobic and resistance; Intensity: Titrated over the first 3–4 weeks to reach 80% HRmax. In resistance training RPE was used	CD34^+^/KDR^+^ (% lymphocytes)		No change in EPC levels. EX group: Pre 0.59 ± 0.38 vs Post 0.49 ± 0.41, *P* > 0.05. Control group: Pre 0.53 ± 0.41 vs Post 0.48 ± 0.49, *P* > 0.05. ↑ FMD (EX group); ↑ Exercise capacity (METs) (EX group); ↔ hs-CRP
Van Craenenbroeck et al. (2010a)	Non-RCT	EX group: *n* = 21 CHF, NYHA class II with LVEF ≤ 40%, 86%males, 61.3 ± 2.2 years, $V{\text{O}}_{{2{\text{peak}}}}$: 18.3 ± 1.4 ml kg^−1^ min^−1^. Control group: *n* = 17 CHF, NYHA class II with LVEF ≤ 40%, 71%males, 63.4 ± 3.0 years, 21.3 ± 2.14 ml kg^−1^ min^−1^	Length: 6 months; Frequency: 3×/week; Duration: 60 min: Modality: Aerobic and (depending on patients’ condition) dynamic resistive exercise incorporated; Intensity: 90%HR at RCP	CD34^+^/KDR^+^/CD3^−^ (Cells/10^6^ events)		Significant improvement in EPCs in the EX group only. EX group: Pre 90 ± 26 vs Post 167 ± 29, *P* = 0.021.Control group: Pre 138 ± 38 vs Post 192 ± 47, *P* = 0.4. ↑ FMD (EX group); ↔ $V{\text{O}}_{{2{\text{peak}}}}$ (both groups); ↔ SDF-1a (both groups)
Trials that combined MICON exercise and calisthenics						
Cesari et al. (2009)	Single arm	EX group: *n* = 86 patients with Cardiac surgery (43% CABG, 57% Valve replacement), 59%males, 72.5 (47–88) years. 6MWT: 304 (53–560) m	Length: 15 days; Frequency: 6x/week (total 12 sessions); Duration: not reported; Modality: Aerobic and short lasting (1-2 min) calisthenic exercises, with the resistance sequentially provided by the weight of single body segments and gentle, passive stretching involving all the main joints; Intensity: Cycling based on HR data from 6MWT	CD34^+^/KDR^+^ and CD133^+^/KDR^+^ and CD34^+^/CD133^+^/KDR^+^ (Cells/μL)	No change on any EPC phenotype group but a better improvement in 6MWT (> 23%) lead to higher number in EPCs after exercise training. CD34^+^/KDR^+^: Pre 0.27 (0–1.65) vs Post 0.26 (0–3.92), *P* > 0.05. CD133^+^/KDR^+^: Pre 0.24 (0–1.18) vs Post 0.25 (0–1.66), *P* > 0.05. CD34^+^/CD133^+^/KDR^+^: Pre 0.16 (0–1.58) vs Post 0.16 (0–1.43), *P* > 0.05. ↑ 6MWT; ↓ VEGF; ↓ hs-CRP. Relationship in patients with 6MWT (> 23%) on CD34^+^/KDR^+^ and VEGF: *r* = 0.37, *P* = 0.01. CD133^+^/KDR^+^ and VEGF: *r* = 0.39, *P* = 0.01. CD34^+^/CD133^+^/KDR^+^ and VEGF: *r* = 0.39, *P* = 0.01	
Gagliardi et al. (2016)	RCT	EX group: *n* = 10 stable CAD patients, 70%males, 59.5 ± 2.8 years, training status: not reported. Control group: *n* = 11, 91%males, 65.4 ± 1.6 years, training status: not reported	Length: 12 weeks; Frequency: 3×/week (2 supervised + 1 home); Duration: not specified; Modality: calisthenics, biking with and without workload, gym or recreational activity, and long walks especially designed for each patient Intensity: not reported	CD34^+^/KDR^+^ (Cells/10^5^ events)	No change in EPC levels. EX group: Pre 0.141 ± 0.022 vs 1 month 0.033 ± 0.004, *P* > 0.05. Pre 0.141 ± 0.022 vs 3 months 0.101 ± 0.037, *P* > 0.05. Control group: Pre 0.208 ± 0.060 vs 1 month 0.041 ± 0.003, *P* > 0.05. Pre 0.208 ± 0.060 vs 3 month 0.088 ± 0.013, *P* > 0.05. Significant inverse correlation between delta EPCs and delta VEGF at 1 month of intervention (ΔEPCs/ΔVEGF: *r* = – 0.57, *P* = 0.007)	
Sandri et al. (2016)	RCT	EX group ≤ 55 years: *n* = 15 CHF patients NYHA II-III with LVEF ≤ 40%, 80%males, 50 ± 5 years, $V{\text{O}}_{{2{\text{peak}}}}$: 13.3 ± 1.6 ml kg^−1^ min^−1^. EX group ≥ 65 years: *n* = 15 CHF patients NYHA II-III with LVEF ≤ 40%, 80%males, 72 ± 4 years, $V{\text{O}}_{{2{\text{peak}}}}$:12.9 ± 1.4 ml kg^−1^ min^−1^. Control group ≤ 55 years: *n* = 15 CHF patients NYHA II-III with LVEF ≤ 40%, 87%males, 49 ± 5 years, $V{\text{O}}_{{2{\text{peak}}}}$:13.6 ± 1.3 ml kg^−1^ min^−1^. Control group ≥ 65 years: *n* = 15 CHF patients NYHA II-III with LVEF ≤ 40%, 80%males, 72 ± 3 years, $V{\text{O}}_{{2{\text{peak}}}}$:13.1 ± 1.5 ml kg^−1^ min^−1^	Length: 4 weeks; Frequency: 4x/week; Duration: 20 min + 1x/week 60 min group session; Modality: Aerobic, calisthenics, balls games; Intensity: Cycling at 70%$V{\text{O}}_{{2{\text{peak}}}}$	CD34^+^/KDR^+^ and CD133^+^/KDR^+^ (Cells/mL)	Significant improvement in EPCs in EX CHF groups only. CD34^+^/KDR^+^: EX group CHF ≤ 55 years: Pre 81 ± 23 vs Post 184 ± 34, *P* < 0.05. EX group CHF ≥ 65 years: Pre 75 ± 24 vs Post 172 ± 39, *P* < 0.05. Control group CHF ≤ 55 years: Pre 90 ± 19 vs Post 93 ± 26, *P* > 0.05. Control group CHF ≥ 65 years: Pre 81 ± 16 vs Post 93 ± 27, *P* > 0.05. CD133^+^/KDR^+^: EX group CHF ≤ 55 years: Pre 72 ± 19 vs Post 169 ± 28, *P* < 0.05. EX group CHF ≥ 65 years: Pre 83 ± 26 vs Post 193 ± 25, *P* < 0.05. Control group CHF ≤ 55 years: Pre 80 ± 27 vs Post 78 ± 25, *P* > 0.05. Control group CHF ≥ 65 years: Pre 87 ± 24 vs Post 83 ± 22, *P* > 0.05. ↑ FMD; (EX groups); ↑ VEGF; (EX groups); ↑ SDF-1a (EX groups)	
Trials that combined MICON exercise and HIIT						
Jo et al. (2020)	Randomised trial	HIIT group: *n* = 17 Hypertensive metabolic syndrome, 70%males, 49.9 ± 7.3 years, training status: not reported. MICON group: *n* = 17 Hypertensive metabolic syndrome, 35%males, 51.8 ± 8.5 years, training status: not reported	Length: 8 weeks; Frequency: 3×/week: Duration: HIIT:35 min, MICON: 35 min; Modality: Interval, Aerobic both on treadmill; Intensity: HIIT: 5 sets of 3 min at 80%HRR with 3 min at 40% of HRR, MICON: 60% at HRR	CD34^+^/KDR^+^ (% Lymphocytes)	~ 88% increase in HIIT group only. HIIT group: Pre 0.017 ± 0.026 vs Post 0.032 ± 0.034, *P* < 0.01. MICON group: Pre 0.029 ± 0.023 vs Post 0.061 ± 0.115, *P* > 0.05. ↑ FMD (in both groups but greater improvements in HIIT group); ↑ NOx (HIIT group only)	
Van Craenenbroeck et al. (2015)	Randomised trial	HIIT group: *n* = 100 stable CAD patients, 91%males, 57 ± 8.8 years, $V{\text{O}}_{{2{\text{peak}}}}$: 23.3 ± 5.8 ml kg^−1^ min^−1^. MICON group: *n* = 100 stable CAD patients, 89%males, 59.9 ± 9.2 years, $V{\text{O}}_{{2{\text{peak}}}}$:22.2 ± 5.6 ml kg^−1^ min^−1^	Length: 12 weeks; Frequency: 3×/week; Duration: HIIT:38 min, MICON: 37 min; Modality: Interval, Aerobic; Intensity: HIIT: 4 min at 90–95%HR_peak_ with 3 min at 50–60% of HR_peak._ MICON: 70–75% at HR_peak_	CD34^+^/KDR^+^/CD45^dim^ (Cells/10^6^ MNCs)	No change in EPC numbers in any of the two exercise groups. HIIT group: Pre 8.2 (0–51) vs Post 7.4 (0–53), *P* > 0.05. MICON group: Pre 9.5 (0–37) vs Post 10.6 (0–106), *P* > 0.05. ↑ $V{\text{O}}_{{2{\text{peak}}}}$ (both groups);↑ FMD (both groups)	
Trials that compared HIIT and combined exercise training						
Kourek et al. (2020a)	Randomised trial	HIIT group: *n* = 21 CHF patients NYHA II-III with LVEF ≤ 49%, 81%males, 56 ± 11 years, $V{\text{O}}_{{2{\text{peak}}}}$:18.7 ± 5.0 ml kg^−1^ min^−1^. COM group: *n* = 23 CHF patients NYHA II-III with LVEF ≤ 49%, 78.3%males, 57 ± 9 years $V{\text{O}}_{{2{\text{peak}}}}$:18.2 ± 3.8 ml kg^−1^ min^−1^	Length: 12 weeks; Frequency: 3×/week (total 36 sessions); Duration: not specified; Modality: Interval (HIIT using cycle ergometry in both groups): Intensity: Workload on HIIT increased progressively to reach + 25% by the end of the programme, HIIT group: 4 min at 80% of $V{\text{O}}_{{2{\text{peak}}}}$ with 3 min at 50% of $V{\text{O}}_{{2{\text{peak}}}}$ + balance and coordination exercises, COM group: HIIT + resistance (2–3 sets,10–12 repetitions at 60–70%1RM with 1 min rest, knee extension, knee flexion and chest press)	CD34^+^/CD45^−^/CD133^+^/KDR^+^ and CD34^+^/CD133^+^/KDR^+^ and CD34^+^/CD45^−^/CD133^−^/KDR^+^ (Cells/10^6^ enucleated cells)	Significant ↑ in all three EPC phenotypes in both training groups with any statistical difference between them. CD34^+^/CD45/CD133^+^/KDR^+^: HIIT group: Pre 2 (1–2) vs Post 6 (5–8), *P* < 0.001.COM group: Pre 2 (1–4) vs Post 4 (3–8), *P* < 0.001. CD34^+^/CD133^+^/KDR^+^: HIIT group: Pre 10 (7–16) vs Post 22 (14–39), *P* < 0.05. COM group: Pre 14 (8–18) vs Post 24 (15–40), *P* < 0.01. CD34^+^/CD45^−^/CD133^−^/KDR^+^: HIIT group: Pre 1 (1–2) vs Post 4 (3–8), *P* < 0.001. COM group: Pre 1 (1–3) vs Post 5 (3–8), *P* < 0.01. ↑ $V{\text{O}}_{{2{\text{peak}}}}$ (both groups); ↑ VEGF (both groups); ↓ CRP (both groups). No relationship between VEGF with any EPC phenotype	

*ADMA* asymmetric dimethyl arginine, *CABG* coronary artery bypass grafting, *CAD* coronary artery disease, *CHF* chronic heart failure, *CPET* cardiopulmonary exercise test, *COM* combined exercise, *GM-CSF* granulocyte macrophage colony stimulating factor, *CRP* C-reactive protein, *CVRF* cardiovascular risk factors, *EPCs* endothelial progenitor cells, *EX* exercise, *FMD* flow-mediated dilatation, *HIIT* high intensity interval training, *HR*_*peak/max*_ Peak/Max heart rate, *hs-CRP*, high sensitivity C-reactive protein, *IQR* interquartile range, *LVEF* left ventricular ejection fraction, *Max.WD* maximum walking distance, *METS* metabolic equivalents, *MICON* moderate intensity continuous exercise, *MMP-2/9* matrix metalloproteinase 2/9, *ml* millilitre, *MNCs* mononuclear cells, *NOx* nitric oxide metabolites (nitrite/nitrate), *NYHA* New York Heart Association functional classification, *non-RCT* non-randomised control trial, *NSTEMI* non ST elevation myocardial infraction, *PAD* peripheral arterial disease, *RCP* respiratory compensation point, *RCT* randomised control trial, *revs/min* revolutions per minute, *SDF-1α* stromal-cell derived factor 1 alpha, *STEMI* ST elevation myocardial infraction, *TIMP-1* tissue inhibitor of metalloproteinase 1, *TNF-α* tumour necrosis factor 1 alpha, *VAT* ventilatory anaerobic threshold, *VEGF-A* vascular endothelial growth factor, *VT1* first ventilatory threshold, *VO*_*2peak/max*_ peak/max oxygen uptake, *1RM* one repetition maximum, *6MWT* six-minute walk test, ↑ indicates significant increase, ↓ indicates significant decrease, ↔ indicates no significant change

Of the 23 chronic clinical trials, 13 trials utilised MICON exercise (cycling or walking) (Sandri et al. 2005; Steiner et al. 2005; Paul et al. 2007; Sarto et al. 2007; Erbs et al. 2010; Schlager et al. 2011; Gatta et al. 2012; Cesari et al. 2013; Eleuteri et al. 2013; Mezzani et al. 2013; Dopheide et al. 2016b), seven investigated a MICON protocol combined with either resistance exercise (Laufs et al. 2004; Van Craenenbroeck et al. 2010b; Hansen et al. 2011; Luk et al. 2012) or calisthenics (Cesari et al. 2009; Gagliardi et al. 2016; Sandri et al. 2016), two compared MICON vs HIIT (Van Craenenbroeck et al. 2015; Jo et al. 2019) and one compared HIIT vs HIIT combined with resistance exercise (Kourek et al. 2020a). The intervention duration ranged from two to 32 weeks, with weekly training frequency two to seven times per week and session duration from 10 to 60 min.

Eight different EPC phenotypes were identified for the quantification of circulating EPCs by flow cytometry. The most common phenotype used was CD34^+^/KDR^+^ (13 trials) (Sandri et al. 2005, 2016; Cesari et al. 2009, 2013; Erbs et al. 2010; Hansen et al. 2011; Gatta et al. 2012; Luk et al. 2012; Gagliardi et al. 2016; Jo et al. 2020), followed by CD34^+^/KDR^+^/CD133^+^ (6 trials) (Steiner et al. 2005; Cesari et al. 2009, 2013; Schlager et al. 2011; Hansen et al. 2011; Kourek et al. 2020a), CD34^+^/KDR^+^/CD45^dim^ (4 trials) (Eleuteri et al. 2013; Mezzani et al. 2013; Van Craenenbroeck et al. 2015; Dopheide et al. 2016b), CD133^+^/KDR^+^ (4 trials) (Paul et al. 2007; Cesari et al. 2009, 2013; Sandri et al. 2016), CD34^+^/KDR^+^/CD31^+^ (1 trial) (Sarto et al. 2007), and CD34^+^/KDR^+^/CD3^−^ (1 trial) (Van Craenenbroeck et al. 2010b). One trial used two versions of four antibody combinations including CD34^+^/CD45^−^/CD133^+^/KDR^+^ or CD34^+^/CD45^−^/CD133^−^/KDR^+^ (Kourek et al. 2020a). The units of measure for EPCs were also variable. Nine trials reported absolute values (cells/mL or cells/μL) (Sandri et al. 2005, 2016; Paul et al. 2007; Sarto et al. 2007; Cesari et al. 2009; Erbs et al. 2010; Hansen et al. 2011), three trials percentage of mononuclear cells (Schlager et al. 2011; Eleuteri et al. 2013; Mezzani et al. 2013), three trials percentage of positive cells (Steiner et al. 2005; Gatta et al. 2012; Dopheide et al. 2016b), two trials cells per 10^6^ events (Van Craenenbroeck et al. 2010b; Cesari et al. 2013), two trials cells per 10^5^ events (Laufs et al. 2004; Gagliardi et al. 2016), one trial cells per 10^6^ mononuclear cells (Van Craenenbroeck et al. 2015) and one trial cells per 10^6^ enucleated cells (Kourek et al. 2020a). Lastly, of the 23 chronic trials, nine trials examined the number and/or function of cultured MACs defined as Di-acLDL ^+^ /lectin ^+^ cells (Laufs et al. 2004; Sandri et al. 2005, 2016; Sarto et al. 2007; Erbs et al. 2010; Van Craenenbroeck et al. 2010b; Schlager et al. 2011) (Supplementary Table S3).

Seventy-eight percent of the trials (*n* = 18) included two blood sampling time points (pre- and post-intervention), three trials five time points (Sandri et al. 2005), and two trials three time points (Schlager et al. 2011; Gagliardi et al. 2016). The post-training blood samples were drawn 48 h after the last training session in five trials (Steiner et al. 2005; Paul et al. 2007; Sarto et al. 2007; Cesari et al. 2009, 2013), 24 h in one trial (Gatta et al. 2012), 24—48 h in one trial (Gagliardi et al. 2016), 72 h in three trials (Sandri et al. 2005), and between 3 – 7 days in one trial (Van Craenenbroeck et al. 2015). Eleven trials did not provide any information regarding timing of blood collection (Table 4) (Laufs et al. 2004; Erbs et al. 2010; Van Craenenbroeck et al. 2010b; Hansen et al. 2011; Luk et al. 2012; Eleuteri et al. 2013; Mezzani et al. 2013; Dopheide et al. 2016b; Sandri et al. 2016; Jo et al. 2020; Kourek et al. 2020a). Blood samples in 13 trials were drawn in a fasted state (Steiner et al. 2005; Cesari et al. 2009, 2013; Erbs et al. 2010; Van Craenenbroeck et al. 2010b, 2015; Hansen et al. 2011; Schlager et al. 2011; Gatta et al. 2012; Luk et al. 2012; Eleuteri et al. 2013; Dopheide et al. 2016b; Jo et al. 2020), while ten trials reported no information regarding fasting status (Table 4) (Laufs et al. 2004; Sandri et al. 2005, 2016; Paul et al. 2007; Sarto et al. 2007; Mezzani et al. 2013; Gagliardi et al. 2016; Kourek et al. 2020a).

**Table 4:** Summary of blood collection time points and in fasting and non-fasting state in chronic trials

Study	Number of blood samples	Time point of blood collection	Fasting/non-fasting status
Chronic clinical trials			
Cesari et al. (2009)	2	Pre and Post intervention (48 h after the last training session)	Overnight fast
Cesari et al. (2013)	2	Pre and Post intervention (48 h after the last training session)	Overnight fast
Dopheide et al. (2016b)	2	Pre and Post intervention	12 h fasting
Eleuteri et al. (2013)	2	Pre and Post intervention	12 h fasting
Erbs et al. (2010)	2	Pre and Post intervention	Fasting
Gagliardi et al. (2016)	3	Pre, 1 month and Post intervention (24-48 h after the last training session)	Not reported
Gatta et al. (2012)	2	Pre and Post intervention for Exercise group only (24 h after the last training session)	Overnight fast
Hansen et al. (2011)	2	Pre and post intervention	Overnight fast
Jo et al. (2020)	2	Pre and Post intervention	8 h overnight fast
Kourek et al. (2020a, b)	2	Pre and Post intervention	Not reported
Laufs et al. (2004)	2	Pre and Post intervention	Not reported
Luk et al. (2012)	2	Pre and Post intervention	Fasting
Mezzani et al. (2013)	2	Pre and Post intervention	Not reported
Paul et al. (2007)	2	Pre and Post intervention (48 h after the last training session)	Not reported
Sandri et al. (2016)	2	Pre and Post intervention	Not reported
Sandri et al. (2005)	5	Baseline, in weeks 1, 2, 3 and post intervention (≥ 72 h resting of physical inactivity)	Not reported
Sarto et al. (2007)	2	Pre and Post intervention (48 h after the last training session)	Not reported
Schlager et al. (2011)	3	Baseline, 3 months and Post intervention (3 days after the last training session)	Fasting
Steiner et al. (2005)	2	Pre and Post intervention (48 h after the last training session)	Fasting
Van Craenenbroeck et al. (2010b)	2	Pre and Post intervention	Overnight fast
Van Craenenbroeck et al. (2015)	2	Pre and Post intervention (3–7 days after the last training session)	Overnight fast

### Quality assessment

Eighteen trials were assessed with the TESTEX scale (Sandri et al. 2005, 2016; Steiner et al. 2005; Erbs et al. 2010; Van Craenenbroeck et al. 2010b, 2015; Hansen et al. 2011; Schlager et al. 2011; Luk et al. 2012; Eleuteri et al. 2013; Mezzani et al. 2013; Dopheide et al. 2016b; Gagliardi et al. 2016; Waclawovsky et al. 2016; Jo et al. 2020; Kourek et al. 2020a). The mean TESTEX score was 9.5 out of 15 (standard deviation 2.8), with a range of 5 – 13 (Table 5). Most of the trials did not report allocation concealment and activity monitoring in control group. Intention to treat analysis was not considered in any of the included trials.

**Table 5:** Quality assessment scores of the randomised controlled trials (RCTs) and non-randomised controlled trials (non-RCTs) according to the TESTEX scale

	Study quality criteria					Study reporting criteria							TESTEX score (out of 15)
	Eligibility criteria specified	Randomisation specified	Allocation concealed	Groups similar at baseline	Blinding of assessor	Outcome measures assessed in 85% of patients	Intention to treat analysis	Between group statistical comparisons reported	Point measures and measures of variability for all reported outcome measures	Activity monitoring in control groups	Relative exercise intensity remained constant	Exercise volume & energy expenditure	
RCTs													
Sandri et al. (2016)	1	1	1	1	1	3	0	2	1	0	1	1	13
Hansen et al. (2011)	1	1	1	0	1	2	0	2	1	1	0	1	12
Luk et al. (2012)	1	1	1	1	1	2	0	2	1	0	1	1	12
Schlager et al. (2011)	1	1	1	1	1	2	0	2	1	0	1	1	12
Waclakovsky et al. (2016)	1	1	0	0	1	3	0	2	1	1	1	1	12
Erbs et al. (2010)	1	1	0	1	1	3	0	1	1	0	1	1	11
Mezzani et al. (2013)	1	1	0	1	1	2	0	2	1	0	1	1	11
Van Craenenbroeck et al. (2015)	1	1	1	1	1	2	0	2	1	0	0	1	11
Eleuteri et al. (2013)	1	0	0	1	0	3	0	2	1	0	1	1	10
Kourek et al. (2020a)	1	1	1	1	0	1	0	2	1	0	1	1	10
Jo et al. (2020)	1	0	0	0	0	1	0	2	1	1	0	1	8
Sandri et al. (2005)	1	0	0	1	0	1	0	2	1	0	1	1	8
Sandri et al. (2005)	1	0	0	1	0	1	0	2	1	0	1	1	8
Sandri et al. (2005)	1	0	0	1	0	1	0	2	1	0	1	1	8
Gagliardi et al. (2016)	1	0	0	1	0	1	0	2	1	0	0	0	6
Non RCTs													
Van Craenenbroeck et al. (2010a, b)	1	0	0	1	0	2	0	2	1	0	0	0	6
Dopheide et al. (2016b)	1	0	0	0	0	1	0	2	1	0	0	0	5
Steiner et al. (2005)	1	0	0	1	0	1	0	1	1	0	0	0	5

The acute trials that included independent groups were assessed with the observational cohort and cross-sectional studies appraisal tool (Table 6). Two acute trials were classified as “good” (Kazmierski et al. 2015; Gevaert et al. 2019), ten as “fair” (Adams et al. 2004; Van Craenenbroeck et al. 2009; Van Craenenbroeck et al. 2010a; Van Craenenbroeck et al. 2011; Rummens et al. 2012; Scalone et al. 2013; Rocha et al. 2015; West et al. 2015; Lutz et al. 2016; Kourek et al. 2020b) and one as “poor” (Shaffer et al. 2006). The majority of the trials received a “No” in sample size justification, different levels of exposure and repeated exposure of assessment questions respectively.

**Table 6:** Quality assessment of observational cohort and cross-sectional trials

Study	Research question	Specified inclusion criteria	Participation ≥ 50%	Uniform eligibility criteria	Sample size	Exposure assessment prior to outcome measure	Sufficient timeframe for effect	Different levels of the exposure of interest	Exposure measure and assessment	Repeated exposure assessment	Outcome measures	Blinding	Follow up rate	Statistical analyses	Quality rating
Gevaert et al. (2019)	Y	Y	Y	Y	Y	Y	N	N	Y	N	Y	CD	NA	Y	Good
Kazmierski et al. (2015)	Y	Y	Y	Y	N	Y	Y	N	Y	N	Y	CD	Y	Y	Good
Adams et al. (2004)	Y	Y	Y	CD	N	Y	Y	N	Y	N	Y	CD	Y	N	Fair
Kourek et al. (2020b)	Y	Y	Y	Y	N	Y	N	N	Y	N	Y	CD	NA	Y	Fair
Lutz et al. (2016)	Y	Y	Y	CD	N	Y	N	N	Y	N	Y	Y	Y	Y	Fair
Rocha et al. (2015)	Y	Y	Y	CD	Y	Y	N	N	Y	N	Y	CD	NA	N	Fair
Rummens et al. (2012)	Y	Y	Y	N	CD	Y	N	N	Y	N	Y	CD	NA	Y	Fair
Scalone et al. (2013)	Y	Y	Y	N	N	Y	Y	N	Y	N	Y	Y	Y	N	Fair
Van Craenenbroeck et al. (2009)	Y	Y	Y	Y	N	Y	N	N	Y	N	Y	CD	NA	Y	Fair
Van Craenenbroeck et al. (2010a)	Y	Y	Y	Y	N	Y	N	N	Y	N	Y	CD	NA	Y	Fair
Van Craenenbroeck et al. (2011)	Y	Y	Y	CD	N	Y	Y	N	Y	N	Y	CD	NA	Y	Fair
West et al. (2015)	Y	Y	Y	CD	N	Y	Y	N	Y	N	Y	CD	NA	Y	Fair
Shaffer et al. (2006)	Y	Y	Y	N	N	Y	N	N	Y	N	Y	CD	N	N	Poor

*CD* cannot decide, *N* no, *NA* not applied, *Y* yes

Seven single arm trials assessed with the Before-After (Pre-Post) studies with no control group appraisal tool (Table 7). Of those, six were classified as “good” (Paul et al. 2007; Sarto et al. 2007; Cesari et al. 2009, 2013; Sandri et al. 2011; Gatta et al. 2012) and one as “fair” (Laufs et al. 2004). The blinding of assessors could not be determined in the majority of the trials, with in only one trial being clearly described (Sarto et al. 2007).

**Table 7:** Risk of bias for before-after (Pre-Post) trials with no control group

Study	Clear research question	Specified inclusion criteria	Participants representative	Enrolment of all eligible participants	Sample size	Clear description of intervention	Definition, validity, reliability of outcome measures	Blind outcome assessment	Follow up rate	Statistical analysis	Multiple outcome measures	Group level intervention & individual outcome effort	Quality rating
Cesari et al. (2009)	Y	Y	Y	Y	Y	Y	CD	CD	Y	Y	NA	NA	Good
Cesari et al. (2013)	Y	Y	Y	Y	CD	Y	CD	CD	Y	Y	NA	NA	Good
Gatta et al. (2012)	Υ	Υ	Υ	CD	CD	Y	Y	CD	Y	Y	NA	NA	Good
Paul et al. (2007)	Y	Y	Y	CD	Y	Y	Y	CD	Y	Y	NA	NA	Good
Sandri et al. (2011)	Y	Y	Y	Y	CD	Y	Y	CD	Y	Y	NA	NA	Good
Sarto et al. (2007)	Y	Y	Y	Y	Y	Y	Y	Y	Y	Y	NA	NA	Good
Laufs et al. (2004)	Y	N	CD	CD	CD	Y	CD	CD	Y	Y	NA	NA	Fair

*CD* cannot decide, *NA* not applied, *N* no, *Y* yes

### Acute effects on circulating EPCs and angiogenic factors

Table 2 shows the acute effects in circulating EPCs and angiogenic factors. When examining the acute effects using CPET on a cycle ergometer two trials found no changes in circulating EPCs in both the CHF patients and healthy control groups (Van Craenenbroeck et al. 2009, 2010a; Gevaert et al. 2019). However, two of those trials found that the migratory capacity of Di-acLDL + /lectin + MACs towards VEGF and SDF-1α was significantly improved after exercise (Van Craenenbroeck et al. 2009, 2010a). In addition, the improvement in MACs migratory capacity was higher (52%) in more severe CHF patients compared to those with mild-CHF (31%), whereas a small reduction was observed in the aged match healthy control group (Van Craenenbroeck et al. 2010a). Another trial found CHF patients (HFrEF and HFmrEF) irrespective of their disease severity based on median $V{\text{O}}_{{2{\text{peak}}}}$ (≥ 18 mL kg^−1^ min^−1^ or < 18 mL kg^−1^ min^−1^), median minute ventilation—carbon dioxide production relationship (VE/VCO_2_ slope) (≥ 32.5 or < 32.5) or left ventricular ejection fraction (≥ 40% or < 40%) were able to mobilise circulating CD34^+^/CD45^−^CD133^+^/KDR^+^ and CD34^+^/CD45^−^/CD133^−^/KDR^+^ EPCs immediately post-exercise (Kourek et al. 2020b). In the same trial, CHF patients with increased disease severity, based on the aforementioned criteria, were able to mobilise CD34^+^/CD133^+^/KDR^+^ EPCs as well as compared to the group with reduced disease severity (Kourek et al. 2020b). One trial reported an increase in EPCs in both their old and young healthy groups but not in the HFrEF group (Van Craenenbroeck et al. 2011). In another trial, there was an increase in CD34^+^/KDR^+^ and CD34^+^/CD133^−^/KDR^+^ EPCs in both the CAD and the healthy groups (Rummens et al. 2012). However, the increase in CD34^+^/CD133^−^/KDR^+^ EPCs was more prominent in the healthy group (Rummens et al. 2012). Finally, one trial (Adams et al. 2004) reported that a symptom-limited test using a cycle ergometer resulted in a significant elevation of CD34^+^/KDR^+^ EPCs by 164% at 24 h and by 76% at 48 h in an ischaemic CAD group. In the same trial the changes on circulating EPCs were accompanied by a 2.9 ± 0.4-fold and 3.3 ± 0.5-fold increase in Di-acLDL ^+^ /lectin ^+^ MACs at 24 h and 48 h respectively (Adams et al. 2004). Two trials assessed vascular endothelial growth factor (VEGF) with one reporting an increase in an ischaemic CAD group (Adams et al. 2004) and the other trial reported no change either in HFrEF or healthy groups (Van Craenenbroeck et al. 2010a). Circulating levels of stromal-cell derived factor one alpha (SDF-1α) was measured in two trials (Van Craenenbroeck et al. 2010a, 2011), with one showing changes in the mild HFrEF and healthy groups but not in the severe HFrEF group (Van Craenenbroeck et al. 2010a). The second one did not report any changes (Van Craenenbroeck et al. 2011). Finally, one trial measured granulocyte macrophage colony stimulating factor (GM-CSF) and basic fibroblast growth factor, but no changes were reported (Adams et al. 2004).

In the trials that incorporated various stress test protocols on a treadmill, one trial found an increase in both CD34^+^/KDR^+^ and CD133^+^/KDR^+^ EPCs by 212% and 278% respectively; this was in turn accompanied by a 230.6% increase in Di-acLDL ^+^ /lectin ^+^ MACs and a 361% increase in serum VEGF in stable PAD patients, but no changes in GM-CSF and basic fibroblast growth factor (Sandri et al. 2011). In a second trial (Shaffer et al. 2006) no effects were observed in various EPC phenotypes in a group of PAD patients. Nevertheless, significant reductions of CD133^+^/CD34^+^/KDR^+^/CD31^−^ EPCs by 63% were evident in the age-matched healthy group.

From the trials that incorporated a MICON protocol, one trial (Lutz et al. 2016) reported a 23% increase on CD34^+^/KDR^+^ EPCs in a normal glucose tolerance group, while no changes were observed in the impaired glucose tolerance and T2DM groups. In another trial (West et al. 2015) increase in CD34^+^/KDR^+^/CD45^dim^ EPCs after ~ 15 h post-exercise was significant in the healthy group but not in the T1DM group. In a third trial (Rocha et al. 2015) the early MetS group had a significant reduction in CD34^+^/KDR^+^ and CD133^+^/CD34^+^/KDR^+^ EPC levels after a bout of MICON compared with their age-matched healthy control group. The latter group increased matrix metalloproteinase (MMP)-2 levels post-exercise while both groups had an increase in granulocyte colony stimulating factor. Moreover, MMP-9 levels increased significantly post-exercise in the early MetS group. VEGF and GM-CSF remained unchanged in both groups.

Lastly, the only trial (Waclawovsky et al. 2016) that compared a MICON cycling bout with a lower limb resistance protocol reported no effect in T1DM patients. In contrast, the healthy control group had a significant increase of EPCs 10 min after the resistance protocol and a significant reduction after the moderate intensity cycling bout.

### Chronic effects on EPCs, fitness status, angiogenic factors, and endothelial function

Table 3 reports the chronic effects of exercise on EPCs, fitness status, angiogenic factors, and endothelial function in clinical populations. Of the 13 trials that included MICON exercise, 10 found significant increases in EPC levels (Sandri et al. 2005; Steiner et al. 2005; Paul et al. 2007; Sarto et al. 2007; Erbs et al. 2010; Schlager et al. 2011; Gatta et al. 2012; Cesari et al. 2013; Eleuteri et al. 2013; Mezzani et al. 2013), two reported no change (Sandri et al. 2005), and one reported more pronounced and significant reduction in a supervised exercise group compared with the non-supervised exercised group (Dopheide et al. 2016b). In four studies, six trials included a cultivation of MACs  to assess their number and/or their function (Sandri et al. 2005; Sarto et al. 2007; Erbs et al. 2010; Schlager et al. 2011). Di-acLDL ^+^ /lectin ^+^ MACs were analysed in 5 of these trials, with three reporting significant increases in their numbers (Sandri et al. 2005; Sarto et al. 2007; Schlager et al. 2011) and two reporting no change (Sandri et al. 2005). Regarding MACs migratory capacity, one trial reported increase by 107.1% (Erbs et al. 2010) and another, increase at 3 and 6 months respectively (Schlager et al. 2011). Finally, three trials reported improvements of MACs ability to participate in network formation with no change to the controls (Sandri et al. 2005).

All 13 trials reported a significant increase in fitness status after the intervention (Sandri et al. 2005; Steiner et al. 2005; Paul et al. 2007; Sarto et al. 2007; Erbs et al. 2010; Schlager et al. 2011; Gatta et al. 2012; Cesari et al. 2013; Eleuteri et al. 2013; Mezzani et al. 2013; Dopheide et al. 2016b) with two trials reporting a significant positive correlation between the increases in EPCs and $V{\text{O}}_{{2{\text{peak}}}}$ (Cesari et al. 2013; Mezzani et al. 2013). Only four trials assessed endothelial function by FMD (Steiner et al. 2005; Paul et al. 2007; Erbs et al. 2010; Eleuteri et al. 2013) with two trials showing improvements (Erbs et al. 2010; Eleuteri et al. 2013) and the other two no changes in FMD (Steiner et al. 2005; Paul et al. 2007). In one of those trials, there was a trend for in improvements in FMD (*P* = 0.07), which was associated with a strong and significant positive correlation between ΔFMD with ΔEPCs: (*r* = 0.81, *P* < 0.01) (Steiner et al. 2005). With respect to circulating angiogenic factors, nine out of the 13 trials measured serum or plasma VEGF (Sandri et al. 2005; Steiner et al. 2005; Sarto et al. 2007; Erbs et al. 2010; Schlager et al. 2011; Eleuteri et al. 2013; Dopheide et al. 2016b). Five of them reporting a significant increase (Sandri et al. 2005; Steiner et al. 2005; Sarto et al. 2007; Erbs et al. 2010; Dopheide et al. 2016b) where one reported a positive relationship between VEGF and EPCs (*r* = 0.66, *P* < 0.05) (Sandri et al. 2005) and one reported an inverse relationship between individual changes in VEGF with changes in EPCs (*r* = – 0.477, *P* < 0.001). Four trials reported no significant changes in any of the primary outcome measures (Sandri et al. 2005; Schlager et al. 2011; Eleuteri et al. 2013). Three trials evaluated the levels of SDF-1α (Sarto et al. 2007; Erbs et al. 2010; Schlager et al. 2011; Eleuteri et al. 2013), with two of them reporting an increase after the intervention (Sarto et al. 2007; Erbs et al. 2010). Three trials measured GM-CSF levels, which remained unchanged at post-intervention (Sandri et al. 2005). One trial measured angiopoietin 1 (Ang-1) and angiopoietin 2 (Ang-2) with significant increases only in the latter. Furthermore, no significant relationships between EPCs, Ang 1, and Ang 2 were observed (Eleuteri et al. 2013). Only one trial assessed MMPs and found no changes in MMP-2 and MMP-9. Interestingly, there was an increase in the ratio of MMPs over the tissue inhibitor of metalloproteinase 1 (MMP-2/TIMP-1 and MMP-9/TIMP-1) respectively (Gatta et al. 2012).

From the four trials that combined resistance and MICON exercise, two observed significant increases in EPC levels (Laufs et al. 2004; Van Craenenbroeck et al. 2010b), which were paralleled with improvements in MACs migratory capacity by 77% (Van Craenenbroeck et al. 2010b), and a reduced rate of apoptosis (Laufs et al. 2004). Furthermore, exercise capacity was improved in three trials (Laufs et al. 2004; Hansen et al. 2011; Luk et al. 2012), whereas in the fourth one the $V{\text{O}}_{{2{\text{peak}}}}$ was not improved (Van Craenenbroeck et al. 2010b). Two trials investigated endothelial function and found improvements in FMD following completion of the combined protocols (Van Craenenbroeck et al. 2010b; Luk et al. 2012).

Of the three trials (Cesari et al. 2009; Gagliardi et al. 2016; Sandri et al. 2016) that implemented MICON exercise and calisthenics, only one found significant increases in EPC levels, which was also accompanied by increases in in MACs migratory capacity, in FMD and SDF-1α (Sandri et al. 2016). Regarding circulating VEGF levels, one trial found an increase (Sandri et al. 2016) whereas another one did not report any changes (Gagliardi et al. 2016) and one reported significant reduction (Cesari et al. 2009).

In the two trials that compared MICON vs a HIIT protocol (Van Craenenbroeck et al. 2015; Jo et al. 2020), one found no changes to circulating EPCs regardless of the protocol followed, (Van Craenenbroeck et al. 2015), While the second one found an 88% increase in EPCs in the HIIT group. This increase was also accompanied by improvements in FMD and an increase in nitric oxide (NOx) metabolites (Jo et al. 2020). In one of the trials (Van Craenenbroeck et al. 2015), $V{\text{O}}_{{2{\text{peak}}}}$ was increased significantly following completion of both training protocols. Regarding endothelial function, vascular FMD was significantly improved in both the HIIT and MICON groups (Van Craenenbroeck et al. 2015; Jo et al. 2020), with greater improvements in the HIIT group (Jo et al. 2020). One trial, which compared HIIT vs HIIT combined with resistance training in CHF patients, found significant increases in all three EPC subpopulations without any significant differences between the exercise protocols. In both protocols there was a significant improvement in $V{\text{O}}_{{2{\text{peak}}}}$, an increase in circulating VEGF and a reduction in C-reactive protein (CRP) (Kourek et al. 2020a).

## Discussion

The primary aim of this review was to investigate the acute and chronic effects of different exercise modalities on circulating EPCs in patients with CVD and metabolic abnormalities. A secondary aim was to identify putative mechanisms of exercise-induced EPC mobilisation and possible links between EPCs and endothelial function as assessed by FMD and aerobic capacity.

In CHF patients, despite the majority of the research indicating that acute EPC mobilisation in HFrEF and HFpEF patients is blunted following completion of a CPET on a cycle ergometer, despite an improvement in MACs migratory capacity, concrete conclusions cannot be made. On the other hand, EPC mobilisation and enhancement of MACs migratory capacity was evident in ischaemic and revascularized CAD completing either a CPET using cycle ergometry or a maximal treadmill exercise test. In PAD patients following a symptom-limited maximal exercise test were equivocal. In patients with altered metabolic health, such as DM, have an impaired ability to mobilise EPCs following completion of an acute exercise bout when compared with healthy age-matched controls.

In the chronic trials, there was strong support for the utility of different exercise training modes for increasing circulating EPCs, irrespective of the phenotype used for EPC identification, in HFrEF and ACS patients. The increase in circulating EPCs showed to be accompanied by increase in MACs ability to migrate. Results were equivocal in CAD patients whereas PAD patients with greater disease severity benefited the most in terms of EPC mobilisation following completion of a chronic exercise training intervention. Long-term training studies provide support for the superiority of HIIT over MICON regarding EPC mobilisation in hypertensive patients with metabolic syndrome.

Angiogenic factors that were more frequently assessed included VEGF, SDF-1α and GM-CSF with evidence suggesting that exercise may have some positive effects on VEGF and SDF-1α. However, there was limited evidence to suggest that observed significant improvements in FMD and aerobic capacity were associated with EPC mobilisation. Collectively our findings suggest that, regardless of exercise mode, chronic training interventions can be an effective means of improving cardiometabolic health. Finally, there is a need for additional research to confirm the long-term impact of exercise on EPC mobilisation in CAD patients, while HIIT training in the long term may be of greater benefit in hypertensive metabolic syndrome patients.

### Acute effects of exercise on EPCs

In HFrEF patients, EPC mobilisation is mostly impaired following completion of CPET protocols involving cycle ergometry (Van Craenenbroeck et al. 2009, 2010a, 2011). In addition, circulating EPCs did not change in the HFrEF group 48 h post-exercise when compared with either a healthy older or healthy younger group (Van Craenenbroeck et al. 2011). However, only one trial found a significant increase in EPC populations after CPET in CHF patients (Kourek et al. 2020b). However, the latter trial included HFmrEF patients in addition to HFrEF patients, who tend to have a higher mean left ventricular ejection fraction. We postulate that the observation that most trials did not find any increase could be partly due to underlying ischaemic factors that can lead to the exhaustion of progenitor cells from the bone marrow (Kissel et al. 2007). It is noteworthy that HFrEF patients’ baseline EPC levels follow a biphasic pattern: at the early stages of the syndrome, there is an increased number (possibly to counterbalance endothelial injury), while in the more advanced stages of the syndrome there seems to be a depletion of the EPC pool (Valgimigli et al. 2004). Consequently, for patients at the early stages of the syndrome, with an already increased baseline EPC number, exercise may not increase EPCs any further due to the already exhausted bone marrow EPC pool (Van Craenenbroeck et al. 2011). The short duration of CPET protocols (i.e., 8–12 min on average) may not be adequate to optimally stimulate the mobilisation of EPCs since in a recent trial in CHF patients both a HIIT of longer duration (4sets of 4 min at 80%$V{\text{O}}_{{2{\text{peak}}}}$ with 3 min at 50%$V{\text{O}}_{{2{\text{peak}}}}$) and a MICON (50%$V{\text{O}}_{{2{\text{peak}}}}$) protocols matched for total work acutely increased circulating EPC levels immediately after and 40 min post-exercise (Mitsiou et al. 2020). In addition, participants in the CHF group with increased disease severity were able to mobilise CD34^+^/CD133^+^/KDR^+^ EPCs compared to participants in the CHF group with reduced disease severity. In healthy individuals, it has been shown that longer exercise times (30 min at either high or moderate intensity) can elicit increases in CD34^+^/KDR^+^ EPCs while short duration exercise (10 min) at moderate intensity failed to (Laufs et al. 2005). In addition, the disparity in the results between the trials included CHF patients could also be accounted to the methodological differences for the EPC quantification such as the selection of EPC phenotype. For example, the trials that did not find any alterations on circulating EPCs used either CD34^+^/KDR^+^/CD3^−^ or CD34^+^/KDR^+^ antibody combination (Van Craenenbroeck et al. 2010a, 2011, 2009) whereas the trial which find increases in EPCs used CD34^+^/CD45^−^/CD133^+^/KDR^+^, CD34^+^/CD133^+^/KDR^+^ and CD34^+^/CD45^−^/CD133^−^/KDR^+^ antibody combinations (Kourek et al. 2020b). Finally, the beneficial effects on angiogenesis after a maximal CPET could possibly derive indirectly by the improvement in MACs migratory capacity (Van Craenenbroeck et al. 2009, 2010a). MACs are monocyte macrophage derived cells that do not differentiate to endothelial cells but play an important role in angiogenesis by releasing angiogenic factors such as IL-8, MMP-9 and VEGF (Medina et al. 2011; Chambers et al. 2013). It is worth noting that the most severe CHF patients had larger increase in MACs migratory capacity (52%) compared to their mild counterparts (31%) (Van Craenenbroeck et al. 2010a). Those results are confirmed by the same authors who found a positive relationship between the baseline NT-pro BNP levels and the exercise-induced MACs migratory capacity (*r* = 0.258, *P* = 0.001).

It appears that HFpEF patients have an impaired ability to mobilise EPCs in response to maximal exercise (Gevaert et al. 2019); however only one blood sample was drawn 10 min post-exercise (CPET) and therefore, future research is required to assess the time course on EPC mobilisation in HFpEF before concrete conclusions can be made.

In CAD patients, post-exercise the number of EPCs increases regardless of the mode of exercise employed, as this was evidenced following completion of a maximal exercise test on a treadmill (Kazmierski et al. 2015) or a cycle ergometer (Adams et al. 2004; Rummens et al. 2012). Adams et al. (Adams et al. 2004) provided evidence about the role of exercise-induced ischaemia on EPC mobilisation. They showed that only the ischaemic CAD group had an increase in EPC levels (including cultured MACs) with no changes observed in the non-ischaemic CAD and healthy aged-matched groups, respectively. However, two other trials demonstrated that EPCs can also be mobilised in revascularized stable CAD patients after maximal exercise test (Rummens et al. 2012; Kazmierski et al. 2015). This observation suggests that acute maximal-intensity exercise mobilises circulating EPCs in both ischaemic CAD and in CAD patients with a restored myocardial perfusion. Despite these positive findings, exercise-induced EPC mobilisation in CAD patients is either reduced (Rummens et al. 2012) or delayed (Kazmierski et al. 2015) when compared to age-matched healthy controls. This further highlights the role of preventative strategies as well as the need for further research into identifying the optimal exercise modes and exercise doses. These will need to be determined with reference to disease severity to optimise EPC mobilisation. Indeed, increased CAD severity (based on the number of stenotic lesions) negatively affects the exercise-induced EPC mobilisation (Kazmierski et al. 2015). This further highlights the need for personalised exercise prescription based on the severity of the disease.

The trials that investigated the acute effects after a maximal exercise test on a treadmill in PAD patients produced equivocal findings. Sandri et al. (2011) noted an increase in circulating EPCs 24 h after exercise. This was accompanied by an increase in plasma VEGF as well. In contrast, Shaffer et al. (2006) did not observe any change in any of the five EPC phenotypes studied. Possible reasons were that in the latter trial only three out of the 15 patients were able to complete 10 min of exercise and therefore, for most of the cohort, there was insufficient time for the exercise stimulus to provoke changes in EPCs. Considering that Sandri et al. (2011) observed that both CD133^+^/KDR^+^ and CD34^+^/KDR^+^ EPCs and Di-acLDL + /lectin + MACs peaked at 24 h post-exercise, Shaffer et al. (2006) possibly failed to capture any changes of EPCs since the time point of blood post-exercise collection was at 10 min.

Evidence from different exercise regimes exists in patients with metabolic disease. One trial examined the acute effects of circulating EPCs in early MetS in comparison to a healthy aged-matched control (Rocha et al. 2015). After 40 min of cycling at 80% of ventilatory threshold there was no change in circulating EPCs compared to baseline in both groups. Post-exercise EPC levels were significantly lower in the MetS group compared to the healthy controls. VEGF and GM-CSF also remained unchanged. A possible explanation for the findings is the sustained increase of MMP-9 levels both at rest (Goncalves et al. 2009) and post-exercise (Rocha et al. 2015) that characterises MetS patients. MMP-9’s crucial role in homing of EPCs (Huang et al. 2009) possibly led to a liberation of EPCs in the circulation followed by their migration to the sites of endothelial injury which led to reduced numbers in circulation.

Acute exercise-induced EPC mobilisation is also altered in T1DM patients. Neither a MICON protocol (West et al. 2015) nor a lower limb resistance exercise bout (Waclawovsky et al. 2016) led to a change in circulating EPCs compared to healthy aged-matched controls. T1DM patients are characterised by increased inflammation and oxidative stress (Devaraj et al. 2006). West et al. (2015) found an inverse relationship between TNF-a and the ΔEPC 15 h post-exercise (*r* = – 0.766, *P* = 0.005). Previous trials showed that TNF-a has inhibitory effects on EPCs by decreasing ex-vivo cultured EPCs through activation of the p38 MAP kinase pathway (Seeger et al. 2005), increasing EPC apoptosis, reducing inducible nitric oxide synthase and endothelial nitric oxide synthase (eNOS) in cultured EPCs (Chen et al. 2011). Furthermore, it was found that glycated haemoglobin was negatively correlated with the EPC changes 15 h post-exercise.

(*r* = – 0.65, *P* = 0.021) (West et al. 2015). Previously, in a large cohort trial of children with T1DM (*n* = 190), reduced glycated haemoglobin levels were the strongest independent predictor of increase in circulating EPCs after a year of follow up (Hortenhuber et al. 2013). Therefore, there is an argument for the use of strategies to increase exercise levels in an attempt to lower inflammation and TNF-a levels and achieve better glycaemic control; these might reverse the blunted EPC response.

Similarly, the EPC responses of older individuals with T2DM or impaired glucose tolerance were blunted compared to the normal glucose tolerance group (Lutz et al. 2016). The authors proposed that the blunted responses of CD34^+^/VEGFR2^+^ EPCs can be accounted for by the impaired ability to increase VEGFR2^+^ cells since the three groups did not differ in CD34^+^ numbers.

To conclude, further research is required to identify the optimum mode, duration and intensity of exercise that can acutely mobilise circulating EPCs in patients with CHF and PAD considering that the included trials in this systematic review incorporated a maximal exercise test. Furthermore, despite the evidence that maximal exercise mobilises EPCs in patients with CAD regardless of the mode of exercise (i.e., cycling or running), no data yet exist regarding any other exercise intensities or other forms of exercise (e.g. resistance exercise or HIIT). Limited evidence exists regarding the acute effects of exercise in patients with metabolic disease and this area needs further investigation. Finally, more research is required to compare different exercise regimes with several collection time points to capture the time course of EPC level in circulation.

### Chronic effects of exercise on EPCs

The present systematic review provides concrete evidence that MICON type interventions have beneficial effects on circulating EPC levels. In particular, patients with HFrEF compared to PAD and CAD patients benefit more from this type of exercise irrespective of EPC phenotype (CD34^+^/KDR^+^, CD34^+^/KDR^+^/CD45^dim^, CD34^+^/KDR^+^/CD31^+^) and duration of the intervention (3–12 weeks) which is in line with previous meta-analyses (Pearson and Smart 2017; Cavalcante et al. 2019). Moreover, MICON exercise apart from the beneficial effects on circulating EPCs showed to enhance the functional capacity of MACs in CHF patients as well (Sarto et al. 2007; Erbs et al. 2010). In ACS patients, a structured MICON programme as short as 4 weeks can increase circulating EPCs irrespective of EPC phenotype, which is accompanied by improvement in $V{\text{O}}_{{2{\text{peak}}}}$ and reduction in high sensitivity C-reactive protein (Cesari et al. 2013).

In PAD patients there were contrasting findings with two trials reporting an increase (Sandri et al. 2005; Schlager et al. 2011), one a reduction (Dopheide et al. 2016b) and another one no change (Sandri et al. 2005) in circulating EPCs. In the trials that reported increases in EPC levels, disease severity was higher than the two trials that did not find positive changes in EPCs as indicated by the baseline maximum walking distances: median values of 148 m (Sandri et al. 2005) and 101.5 m (Schlager et al. 2011) vs 401 m (Dopheide et al. 2016b) and 335 m (Sandri et al. 2005). A cross-sectional trial found that CD34^+^/VEGFR2^+^/CD45^dim^ EPCs were proportionally higher in patients with increased disease severity (but not critical limb ischaemia CLI) (Dopheide et al. 2016a). On this basis, we propose that PAD patients with greater disease severity (but not CLI) would benefit from MICON exercise programmes due to the ongoing demand for endothelial repair and the augmented EPC response to exercise training.

In CAD patients the effects of a MICON exercise programme are inconclusive with results suggesting that a 4-week programme is not adequate to increase circulating EPCs (Sandri et al. 2005). It is worth noting that in this study, exercise was completed in bouts of 10 min repeated 6 times a day at an intensity of 70% HRpeak. Longer training programmes lasting 12 weeks showed a significant increase in EPC levels, however, this was not paralleled with significant improvement in FMD (Steiner et al. 2005; Paul et al. 2007). In contrast, 12 weeks of a MICON exercise programme increased FMD but not circulating EPCs (Van Craenenbroeck et al. 2015). These variations in findings can partly be explained by individual responses (responders and non-responders) to the given exercise stimulus. Paul et al. (2007) reported that despite the significant increase in circulating EPCs, 46 participants (23.9% of total of sample size) did not show any changes in EPC levels. They found no differences in baseline EPCs, anthropometric characteristics, medical therapy, or other potential parameters that would explain differences in EPC levels between responders and non-responders. This requires further investigation to ascertain the optimal dose of exercise to each patient. Another possible confounder is the (often not reported) training fidelity. In other words, it is assumed (but not monitored) that participants follow the training instructions they have received, in terms of frequency, duration and intensity of exercise, for the duration of the study (Ibeggazene et al. 2020).

Interventions combining resistance with MICON exercise produce variable results regarding EPC responses. HFrEF patients seem to benefit from such type of interventions as evidenced by improvements in FMD (Van Craenenbroeck et al. 2010b). In CAD patients only one trial found increases in EPC levels after 28 days of training (Laufs et al. 2004). In other studies, training for six (Hansen et al. 2011) and eight (Luk et al. 2012) weeks was not accompanied by EPC increases, despite FMD improvement in the latter trial. Several methodological differences contribute to the variability in findings. For example, the two trials which reported improvements in EPC numbers (Laufs et al. 2004; Van Craenenbroeck et al. 2010b) had participants of a lower aerobic capacity at baseline and seem to have prescribed higher exercise doses than the studies which showed no effect (Hansen et al. 2011; Luk et al. 2012) though the lack of detailed descriptions of the exercise interventions makes this hard to quantify. Nevertheless, both volume and intensity seem higher in these trials (Laufs et al. 2004; Van Craenenbroeck et al. 2010b). It is unfortunate that there seems to be a lack of detailed information regarding the resistance exercise prescription (Laufs et al. 2004; Luk et al. 2012), the exact time point of blood collection post-intervention (Laufs et al. 2004; Hansen et al. 2011; Luk et al. 2012) and whether the patients were fasted or not during the blood sampling (Laufs et al. 2004). Considering that EPC levels have been shown to peak at 24 h and remain elevated up to 48 h after an acute exercise bout in CAD patients (Adams et al. 2004), while consumption of beverages such as coffee and beer increase circulating EPCs in CAD and individuals with increased CVD risk respectively (Spyridopoulos et al. 2008; Chiva-Blanch et al. 2014), information about blood time collection and fasting status is essential.

When examining the effects of MICON exercise with the addition of callisthenics only one trial found that both CD34^+^/KDR^+^ and CD133^+^/KDR^+^ EPCs increased in patients with HFrEF irrespective of age (Sandri et al. 2016). The increased EPC numbers were accompanied by improvements in FMD, MACs migratory capacity towards SDF-1α, VEGF and SDF-1α. The lack of positive responses in another trial can be attributed to the short duration intervention (15 days) and the wide range of co-morbidities and cardiovascular risk factors within the cohort (Cesari et al. 2009). For example, cardiac patients with PAD had lower EPC numbers at baseline compared to the patients without, which probably contributed to a heterogeneity in terms of the effects of the exercise programme on EPC levels (Cesari et al. 2009). However, when the cohort was divided based on median percentage improvement in the six-minute walk test (6MWT), the patients with > 23% improvement had significantly increased EPC levels irrespective of the EPC phenotype (CD34^+^/KDR^+^; CD133^+^/KDR^+^; CD34^+^/CD133^+^/KDR^+^) (Cesari et al. 2009). Also, in the same patients, there was a positive relationship between EPC numbers and VEGF whereas in those with < 23% improvement in 6MWT there was a negative correlation between CRP and EPC levels. Moreover, in another trial, CAD patients who combined aerobic activities with callisthenics, despite a lack of changes in EPC numbers, there was an inverse relationship between circulating EPCs and VEGF (*r* = – 0.57, *P* = 0.007) (Gagliardi et al. 2016). The authors attributed this relationship to the increased incorporation of EPCs into sites of endothelial injury leading to reduced levels in circulation (Gagliardi et al. 2016). The above data are inconclusive and further investigation is required regarding the value of adding callisthenics into cardiac rehabilitation programmes alongside aerobic exercise. Nevertheless, evidence suggests that breaks of sedentary time every 20 min with five calisthenic exercises compared to sitting induce an increase in brachial shear rate (Carter and Gladwell 2017). Moreover, a 12 week aerobic calisthenic exercise programme led to an improvement in lipid profile and increase in plasma NOx (Guzel et al. 2012). However, no study to our knowledge investigated the effects of breaking sedentary time on circulating EPC levels. Considering the critical role of increased shear stress and NOx on EPC mobilisation and homing (Obi et al. 2014; Ozuyaman et al. 2005) further investigation is warranted in the use of structured callisthenic exercise programmes in clinical populations.

Three trials investigated the chronic effects of HIIT programmes on EPC levels in comparison to either MICON or combined (HIIT plus resistance exercise) modalities. This follows with contrasting findings as one trial reported significant increases in CD34^+^/KDR^+^ EPCs in the HIIT group (Jo et al. 2020), also accompanied by greater FMD improvements (Jo et al. 2020), whereas the other trial found no effects on CD34^+^/KDR^+^/CD45^dim^ EPCs despite both the HIIT and MICON protocols induced similar improvements in FMD (Van Craenenbroeck et al. 2015). Moreover, addition of resistance exercise to HIIT programmes elicited positive changes on circulating EPCs, which were accompanied by concomitant increases in VEGF and a reduction in CRP (Kourek et al. 2020a). One possible reason for the contrasting findings is the varied pathological status of the participants (hypertensive MetS, CHF and CAD), which can potentially influence to circulating EPC kinetics. Furthermore, two trials were single centre (Jo et al. 2020; Kourek et al. 2020a) whereas the third one was a multicentre trial (Van Craenenbroeck et al. 2015); this may have led to a potential variation in the assessment processes and application of training programmes between centres (Conraads et al. 2015).

In conclusion, strong evidence exists that patients with HFrEF and ACS benefit from a structured exercise programme and see significant increases in circulating EPCs. MICON exercise programmes seems to primarily benefit PAD patients with increased disease severity. In CAD patients the findings are equivocal with the data showing large variability in terms of EPC phenotypes, training characteristics, disease severity and medication prescription. In hypertensive MetS, a single trial showed that a HIIT intervention was superior to a MICON exercise intervention in terms of EPC mobilisation. Only two trials included in this review compared two different exercise protocols against each other. Therefore, more head-to-head RCTs are needed to compare the effects of different exercise protocols on EPC mobilisation. Finally, the chronic effects of different exercise modalities on HFpEF and T1DM and T2DM are yet to be determined. It is also important that studies improve their reporting of patient characteristics (especially in terms of anthropometry and cardiovascular fitness as well as clinical details and medications) and the reporting of details of the exercise interventions (frequency, intensity and duration of exercise as well as type and mode). Additionally, as exercise effects are specific to the above characteristics, an attempt should be made to monitor adherence to the prescribed exercise programme (exercise fidelity) and results should be analysed and presented based both on (a) intention to treat and (b) per protocol.

### Mechanisms for exercise-induced EPC mobilisation

The mechanisms through which exercise mobilises circulating EPCs are not fully understood. VEGF is an important humoral factor that is upregulated in cells exposed to hypoxia or ischaemia via hypoxia-inducible transcription factor 1 alpha (Forsythe et al. 1996). Acutely, a symptom-limited maximal exercise test was shown to increase plasma VEGF levels in ischaemic CAD and PAD patients (Adams et al. 2004; Sandri et al. 2011). These findings were not observed in non-ischaemic CAD patients (Adams et al. 2004) suggesting that transient exercise-induced ischaemia is an important factor in increasing VEGF and consequently circulating EPCs. The lack of increases in other angiogenic factors such as GM-CSF, basic fibroblast growth factor and SDF-1α makes it difficult to fully understand the exact mechanisms responsible for exercise-induced EPC mobilisation after acute exercise in the clinical context.

More evidence exists regarding the mechanisms for EPC mobilisation after chronic exercise. Exercise interventions showed that an increase in EPC levels was accompanied by an increase in VEGF in HFrEF (Erbs et al. 2010; Sandri et al. 2016; Sarto et al. 2007; Kourek et al. 2020a), in CAD (Steiner et al. 2005) and ischaemic PAD (Sandri et al. 2005). This increase in VEGF was found to be positively correlated with EPC levels (*r* = 0.66, *P* < 0.05) (Sandri et al. 2005). Besides, Cesari et al. (2009) found that post-cardiac surgery, in patients who had a > 23% median improvement in 6MWT, VEGF levels were positively correlated with EPC levels. The above indicates that VEGF play a positive role in the mobilisation of EPC into the circulation.

SDF-1α is another putative factor involved in EPC mobilisation via the upregulation of chemokine receptor 4/Janus Kinase-2 signalling (Xia et al. 2012). Three trials reported an elevation in SDF-1α levels which coincided with an increase in circulating EPCs (Erbs et al. 2010; Sandri et al. 2016; Sarto et al. 2007). In contrast, two trials failed to find an increase in SDF-1α despite an increase in EPC levels in the exercise groups (Van Craenenbroeck et al. 2010b; Eleuteri et al. 2013). These findings can be attributed to the difference in disease severity of the HFrEF patients between the trials. SDF-1α levels tend to be elevated in more advanced CHF patients (higher New York Heart Association Classification classes) (Valgimigli et al. 2004). The trials that reported no increases in SDF-1α had patients classified as New York Heart Association Classification II, whereas trials which reported increases in SDF-1α had a large portion of their patients at New York Heart Association Classification class III. Therefore, an exercise intervention can possibly be more beneficial to HFrEF patients with more advanced disease by causing greater increases in SDF-1α to compensate for the diminished EPC levels. Finally, the discrepancy in the results could be accounted to differences in the methodological steps quantifying circulating SDF-1α. The latter is a chemokine ligand to CXCR4 membrane receptor (Thon 2014). CXCR4 expressed on megakaryocyte lineage including platelets (Wang et al. 1998). Incomplete or no removal of platelets during plasma processing could lead to misleading results on SDF-1α levels caused by platelets activation instead of the release to the circulation from the tissues.

NOx bioavailability also plays a crucial regarding EPC mobilisation. The importance of NOx bioavailability on EPCs via eNOS mRNA expression was documented by Laufs et al. (2004) in trained wild type mice. In the present review two trials found that increases in circulating EPCs paralleled increases in NOx levels in HFrEF and hypertensive MetS patients respectively (Steiner et al. 2005; Jo et al. 2020). The increases in NOx levels were strongly correlated with increases in circulating EPCs (*r* = 0.83, *P* < 0.01) (Steiner et al. 2005).

Moreover, decreased EPC levels have been attributed to pro-inflammatory markers such as TNF-α and CRP. TNF-α is known for its myelosuppressive effects on EPC numbers in HFrEF (Valgimigli et al. 2004). In the present review we present evidence that TNF-α levels can be reduced after chronic exercise, which is paralleled by an increase in EPC numbers (Erbs et al. 2010; Gatta et al. 2012). This increase was inversely correlated with TNF-α levels (*r* = – 0.788, *P* < 0.01) (Gatta et al. 2012). It has been shown that incubation of cultured EPCs with SB203580 (a p38-kinase inhibitor) led to increased EPC numbers and diminished the negative effects on them from TNF-α (Seeger et al. 2005). Therefore, chronic exercise, through a reduction of systemic TNF-α levels might act as an inhibitor to the deleterious effects of the latter on EPC numbers and survival. Increased CRP levels are known to increase production of reactive oxygen species in EPCs causing apoptosis and necrosis (Fujii et al. 2006) and inhibit EPC differentiation and function via reduction of EPC eNOS mRNA expression (Verma et al. 2004). Some trials showed that CRP levels are reduced after chronic exercise (Cesari et al. 2009, 2013; Dopheide et al. 2016b; Kourek et al. 2020a), while others not (Paul et al. 2007; Hansen et al. 2011; Luk et al. 2012; Eleuteri et al. 2013). However, Cesari et al. (2013) using a large sample of patients with ACS (*n* = 112) found that baseline hsCRP levels were a significant predictor of increased EPC levels after the cardiac rehabilitation programme.

Finally, asymmetric dimethylarginine is an endogenous NOx inhibitor, which causes endothelial dysfunction and serves as a surrogate marker in CV disease (Sibal et al. 2010). Plasma ADMA is found to be inversely correlated with CD34^+^/CD133^+^ progenitor cells, reduced EPC function in vitro and decreased EPC eNOS activity (Thum et al. 2005). In the present systematic review despite the limited evidence, one RCT showed that asymmetric dimethylarginine levels were decreased at 3 and 6 months of exercise training compared to the control group. This decrease in asymmetric dimethylarginine levels paralleled an increase in CD34^+^/KDR^+^/CD133^+^ EPCs (Schlager et al. 2011).

In most of the trials assessing the chronic effects of exercise, the increase in circulating EPCs has coincided with an improvement in aerobic capacity (e.g., $V{\text{O}}_{{2{\text{peak}}}}$, 6MWT, maximal walking distance) (Laufs et al. 2004; Sarto et al. 2007; Erbs et al. 2010; Adams et al. 2013; Cesari et al. 2013; Eleuteri et al. 2013; Mezzani et al. 2013; Kourek et al. 2020a). However, the role of circulating EPCs in the improvement of aerobic capacity is not clear. A cross-sectional study also found that the haemodialysis patients with various co-morbidities who had higher exercise capacity had higher basal EPC counts (Manfredini et al. 2007). Another recent cohort trial found that CD34^+^ progenitors but not CD34^+^CD133^+^KDR^+^ EPCs were predictors of all-cause and CVD mortality in CVD patients with higher level of physical activity associated with higher CD34 ^+^ progenitors (Muggeridge et al. 2021). To explain any mechanistic link between EPC mobilisation and $V{\text{O}}_{{2{\text{peak}}}}$, one trial did not find any relationship (Eleuteri et al. 2013), while two found a positive relationship in ACS and HFrEF patients respectively (Cesari et al. 2013; Mezzani et al. 2013). Mezzani et al. (2013) suggested EPCs possibly had a positive effect on $V{\text{O}}_{{2{\text{peak}}}}$ by mediating changes in the altered microvasculature which led to an improvement in oxygen utilisation and aerobic power. Certainly, it is possible that improvement in both variables is the product of an appropriate exercise dose and, otherwise, mechanistically unrelated.

**Fig. 2:**
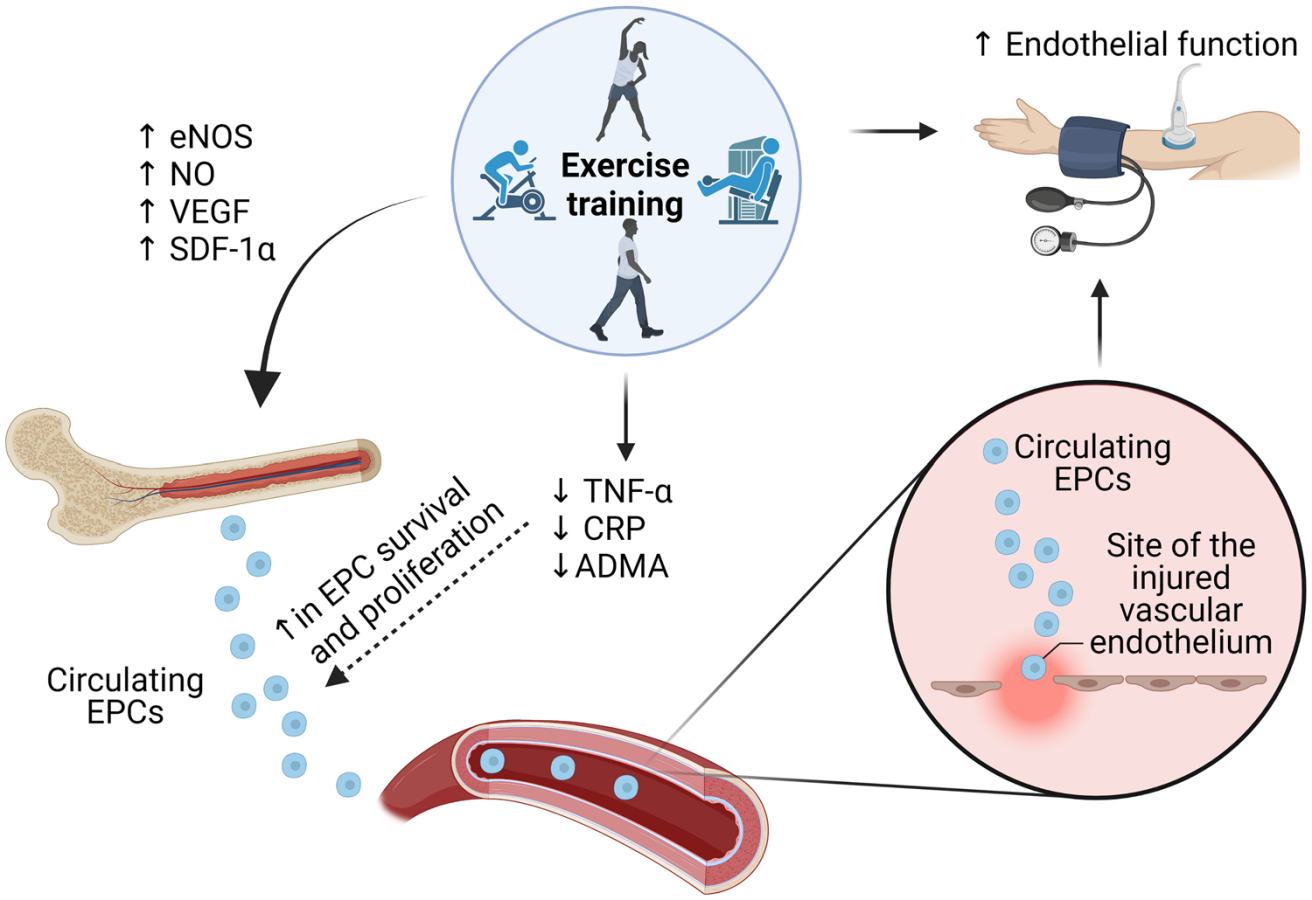
Exercise training induces increases through shear stress to eNOS and consequently to nitric oxide bioavailability. In addition, exercise training increases the levels of pro-angiogenic factors such as VEGF and SDF-1α. EPCs mobilised from the bone marrow niche to the circulation following a gradient of SDF-1α. Long-term exercise leads to reduction to NO inhibitor, ADMA and pro-inflammatory markers such as TNF-α and CRP and consequently increase EPC proliferation and survival. The changes induced by exercise training in EPC levels coincide with improvements in endothelial function

The evidence of the impact of EPCs on macrocirculation as assessed by FMD is currently limited. In the present review, some trials found that the observed increases in circulating EPCs were paralleled by improvement in FMD (Erbs et al. 2010; Van Craenenbroeck et al. 2010b; Eleuteri et al. 2013; Sandri et al. 2016). Steiner et al. (2005) found that the change of EPCs was strongly positively correlated with changes in FMD (*r* = 0.81, *P* < 0.01). In contrast, Paul et al. (2007) did not find improvement in FMD despite an increase in EPC numbers, whereas van Craenenbroeck et al.(2015) reported that HIIT and MICON exercise protocols elicited equal improvements in FMD without any changes in EPC numbers. Furthermore, Luk et al. (2009) demonstrated that in CAD patients increased habitual physical activity levels were significantly correlated only with FMD and not with circulating EPCs suggesting an improvement in endothelial function through other mechanisms and not related to EPCs. Given that circulating EPC numbers and FMD are independent predictors of cardiometabolic diseases (Meyer et al. 2005; Kunz et al. 2006; Guazzi et al. 2009; Sambataro et al. 2014; Koller et al. 2016) further research is required to investigate the potential link between them following an exercise programme. The potential mechanisms of exercise-induced upregulation of EPCs for endothelial repair based on the findings of the current review are illustrated in Fig. 2.

Finally, the exercise-induced EPC mobilisation and the relationship with microcirculation is understudied. Patients with CAD and DM are characterised by altered microvascular function (Borges et al. 2016; Strain and Paldánius 2018),while in CHF patients impaired microcirculation is associated with exercise intolerance (Manetos et al. 2011). In the present systematic review, only a single trial assessed and found that a moderate exercise training intervention led to concomitant improvement in peripheral microcirculation and circulating EPCs in CHF patients (Mezzani et al. 2013), which possibly suggest that circulating EPCs play a healing role to the injured endothelium at the microcirculatory level. More research is warranted about the link between circulating EPCs and microcirculation during exercise interventions that implement different modalities, intensities and durations given that combined (resistance and aerobic) exercise or increased exercise frequency enhances microcirculation in CAD and MetS patients respectively (Borges et al. 2018; Marini et al. 2019).

### Strengths and limitations

This systematic review has several strengths: To our knowledge, this is the first systematic review examining the acute effects along with the chronic effects of different exercise modalities in patients with cardiovascular and metabolic diseases. Previous systematic reviews and meta-analyses focussed only on the chronic effects of physical exercise on circulating EPCs in cardiovascular disease (Pearson and Smart 2017; Cavalcante et al. 2019; Ribeiro et al. 2013). However, they have been either contacted up to 2013 (Ribeiro et al. 2013) or the meta-analysis was based only on CHF patients which included only three trials (Pearson and Smart 2017). In addition, a recent meta-analysis (Cavalcante et al. 2019) focussing on effects of exercise on EPCs in CV disease failed to include two papers that included four RCTs (Sandri et al. 2005; Erbs et al. 2010). In the present systematic review, there was a rigorous application of the systematic review methodology and an extensive searching of the relevant literature. Despite that we did not included only RCTs we used valid quality assessment tools suitable for each study design. Moreover, we provide a detailed information of the different exercise modalities and the related angiogenic factors to delineate the existing literature related to EPC mobilisation in the peripheral blood and the potential exercise-induced mechanisms.

Our study has also some limitations. A limitation of the evidence in this review is the large variability in terms of the methodological assessment used by trials for identification of circulating EPCs. Two main methods have been used to identify EPCs: (a) flow cytometry based on combination of different cell surface markers, (b) in vitro cell culture methods (Medina et al. 2012). In culture, there are two distinct cell populations that differ based on their lineage, characteristics, and progeny. These are MACs and endothelial colony forming cells. In the present systematic review we identified, reported and used the most contemporary nomenclature for MACs, which are not progenitors but angiogenic cells that assist indirectly to vascular repair (Medina et al. 2017). However, it has been questioned whether cultured MACs really do exist in-vivo (Medina et al. 2017; Fadini et al. 2012). In addition, flow cytometry is the most appropriate method to quantify circulating EPCs (Fadini et al. 2008a; Van Craenenbroeck et al. 2013). For the reasons mentioned above we included only the trials that quantified circulating EPCs by flow cytometry as their primary method to provide a more consistent picture in the topic. However, despite flow cytometry being considered the gold standard to quantify EPCs in the blood, there is no standard phenotype that best represents this population (Madonna and De Caterina 2015). In the present review, we identified eleven different EPC phenotypes for the acute trials and eight for the chronic trials which confirm the large heterogeneity between the trials. However, CD34^+^/KDR^+^ or CD34^+^/KDR^+^/CD45^dim^ antibody combination is the best compromise to quantify EPCs in terms of sensitivity, specificity and reliability in the clinical setting (Schmidt-Lucke et al. 2010; Fadini et al. 2012; Van Craenenbroeck et al. 2013). Differences in gating strategies to identify EPCs account to the variability in the present results. Van Craenenbroeck et al. (2008) found moderate to poor agreement between six different flow cytometric gating strategies for EPC quantification. Finally, flow cytometry related analytical factors possibly can explain part of the variability on findings within the same clinical population since the volume of blood samples, storage protocol, anti-coagulant tube selection, a wash step or not for red blood cell lysis and units of EPC expression are essential for reliable and accurate EPC enumeration (Hoymans et al. 2012; Van Craenenbroeck et al. 2013; Zhou et al. 2018).

In the present systematic review, the majority of the trials that investigated the acute effects in EPCs used post-exercise one-time point only for blood sample with the most common being 10 min post-exercise. This potentially masked any increases on circulating EPCs since it has been shown that EPC levels remain elevated after 24 h in ischaemic CAD and PAD patients (Adams et al. 2004; Sandri et al. 2011). Because of the potential delayed acute effects of exercise, it is not known if there was an effect of the last training session in chronic trials since a large number of them did not specify when the sample was taken at the end of the intervention. Moreover, in many acute and chronic trials, the condition of the patients whether were fasted or not during the blood sampling was not clearly stated.

In the present systematic review, we have shown that most of the participants were males (78.5%). Given that circulating EPCs are regulated by sex hormones such as oestrogens (Fadini et al. 2008b) more investigation required in the female population to identify potential different responses to exercise in EPC mobilisation.

All the acute trials that included patients with CV conditions the exercise protocol employed was a symptom-limited maximal exercise test on a treadmill or cycle ergometer. However, this does not represent a real exercise training condition (Volaklis et al. 2013), while the short duration of the test is likely an inadequate stimulus to significantly increase circulation of EPCs. It would therefore be prudent that future trials employ “real life” exercise training protocols based on international guidelines and include several time points to capture the time course of EPC mobilisation.

A recurrent limitation in some of the chronic trials was the lack of information regarding exercise prescription. For example, in many trials there was not provided sufficient information regarding the duration, repetitions or intensity (Laufs et al. 2004; Steiner et al. 2005; Sandri et al. 2005, 2016; Paul et al. 2007; Cesari et al. 2009; Van Craenenbroeck et al. 2010b; Luk et al. 2012; Adams et al. 2013; Gagliardi et al. 2016).

HIIT and whole-body resistance exercise found to be understudied in the present review. For example, whole-body resistance exercise showed to increase circulating EPCs in healthy males and females respectively (Ross et al. 2014; Ribeiro et al. 2017). Moreover, groups of patients such as HFpEF, T1DM and T2DM were understudied in terms of the effects of exercise on circulating EPCs and the angiogenic factors, therefore future trials should focus on these populations.

Finally, one overlooked topic is the interaction between medication and exercise interventions. For example, due to the known beneficial effects of statins (Lee and Poh 2014), changes in statin dosage can hinders the investigation of the effects of exercise on circulating EPCs. In chronic trials with CAD patients only some (Laufs et al. 2004; Steiner et al. 2005; Paul et al. 2007; Luk et al. 2012) clearly stated that the medication did not change throughout the intervention. This observation warrants more investigation because it is not known if it can (at least in part) explain the differences in the results in this patient group.

## Conclusions

This systematic review provides evidence that an acute exercise bout increases EPC numbers as well as cultured MACs in ischaemic and revascularized CAD patients and patients with PAD, where these increases peak at 24 h post-exercise. In contrast, HFpEF have an impaired ability to mobilise EPCs. In HFrEF, inconclusive findings suggest that maximal exercise does not appear to favour EPC mobilisation, despite an increase in MACs migratory capacity. However, the evidence comes only from the use of a maximal exercise test as the exercise stimulus. Some evidence exists that HIIT and MICON exercise modalities acutely enhance EPC numbers. In patients with metabolic diseases EPC mobilisation is blunted compared to healthy matched controls.

In chronic trials, we found evidence that HFrEF and ACS patients benefit from increases in circulating EPCs and MACs migratory capacity as a result of participation in MICON exercise programme alone or with the addition of resistance exercise or group session with callisthenics. These improvements were irrespective of the EPC phenotypes used. PAD patients with reduced physical capacity seem to benefit from a MICON exercise programme. In CAD patients, findings are equivocal possibly due to variation in EPC phenotypes, training characteristics, disease severity and medication. In hypertensive MetS patients, a HIIT protocol seems to be superior for EPC mobilisation and improvement in vascular function.

The effects of exercise on circulating EPCs are related to angiogenic factors such as VEGF, SDF-1α, increase in NOx bioavailability, reduction in inflammation and asymmetric dimethylarginine. The potential relationship with FMD and $V{\text{O}}_{{2{\text{peak}}}}$ is yet to be determined.

Despite the abundance of literature around circulating EPCs and exercise further research is still required to investigate the feasibility and dose–response relationship of various “real life” exercise training protocols, and to assess the time course of EPC release in the circulation.

Finally, head-to-head RCTs are now required to examine and compare various exercise protocols such as HIIT and whole-body resistance exercise in patients with cardiovascular and metabolic disease using standardised methodologies for accurate, valid and reliable EPC enumeration.

## Supplementary Information

Below is the link to the electronic supplementary material.

Supplementary file1 (DOCX 13 KB)

Supplementary file2 (DOCX 18 KB)

Supplementary file3 (DOCX 18 KB)

## Abbreviations

1RM: One repetition maximum strength
6MWT: Six minute walk test
CAD: Coronary artery disease
CHF: Chronic heart failure
CPET: Cardiopulmonary exercise test
CRP: C- reactive protein
CVD: Cardiovascular disease
DM: Diabetes mellitus
eNOS: Endothelial nitric oxide synthase
EPCs: Endothelial progenitor cells
FMD: Flow-mediated dilatation
GM-CSF: Granulocyte macrophage colony stimulating factor
HFpEF: Heart failure preserved ejection fraction
HFrEF: Heart failure reduced ejection fraction
HIIT: High intensity interval training
MACs: Myeloid angiogenic cells
MetS: Metabolic syndrome
MICON: Moderate intensity continuous training
MMP: Matrix metalloproteinase
non-RCTs: Non-randomised controlled trials
NOx: Nitric oxide metabolites (Nitrite/Nitrate)
PAD: Peripheral arterial disease
PRISMA: Preferred reporting items for systematic reviews and meta-analyses
RCT: Randomised controlled trial
SDF-1α: Stromal cell derived factor 1 alpha
T1DM: Type one diabetes mellitus
T2DM: Type two diabetes mellitus
TESTEX: Tool for the assessment of study quality and reporting in exercise
TIMP-1: Tissue inhibitor of metalloproteinase 1
TNF-a: Tumour necrosis factor 1 alpha
VEGF: Vascular endothelial growth factor
