# Supplementary material for: The impact of different forms of exercise on circulating endothelial progenitor cells in cardiovascular and metabolic disease
Source: Eur J Appl Physiol. 2022 Jan 12;122(4):815–60. doi: 10.1007/s00421-021-04876-1 (PMC8927049; doi:10.1007/s00421-021-04876-1)
Supplement: Supplementary file 1 — Supplementary file1 (DOCX 13 KB) [file 421_2021_4876_MOESM1_ESM.docx]

**S1. Research strategy**

**Population:**

“coronary artery disease” OR “acute coronary syndrome” OR “heart failure” OR “type I diabetes mellitus” OR “type II diabetes mellitus” OR “peripheral arterial disease” OR “acute myocardial infarction” OR “myocardial infarction” OR “cardiovascular risk factors” OR “cardiometabolic risk factors” OR “healthy people” OR “healthy subjects” OR “healthy individuals” OR “young people” OR “young healthy” OR “older people” OR “older persons” OR “older individuals” OR “older adults” OR elderly OR aging OR ag?ing OR athletes OR “physically active” OR “active people” OR “inactive people” OR “physically inactive” OR sedentary OR m?n OR wom?n OR adults OR human OR “homo sapiens” OR “modern m?n”

**Exercise**

“physical exercise” OR exercise OR “physical training” OR training OR “physical activity” OR “human physical conditioning” OR “human physical training” OR “chronic exercise” OR “acute exercise” OR “aerobic exercise” OR “moderate intensity exercise” OR “moderate continuous exercise” OR cycling OR “cycling ergometer” OR treadmill OR running OR “resistance exercise” OR “resistance training” OR “strength training” OR “weight-lifting exercise programme” OR “weight bearing strengthening programme” OR “high-intensity interval exercise” OR “high intensity interval training” OR “high-intensity Intermittent exercise” OR “intermittent exercise” OR “sprint Interval training” OR “circuit based exercise” OR “circuit training” OR “strenuous exercise” OR “training programme” OR “exercise intervention” OR “rehabilitation Exercise” OR “exercise therapies” OR “exercise programme” OR “maximal exercise” OR “cardiopulmonary exercise test” OR “stress test” OR “cardiac rehabilitation” OR rehabilitation

**Endothelial progenitor cells**

“endothelial progenitor cells” OR “putative endothelial progenitor cells” OR EPC OR “early EPCs” OR “late EPCs” OR “circulating endothelial progenitor cells” OR “endothelial progenitors” OR “circulating angiogenic cells” OR “circulating progenitor cells” OR “progenitor cells” OR “endothelial progenitor cell mobilization” OR progenitor cell mobilization OR “angiogenic precursors” OR CD34+VEGFR2+ OR CD34+KDR OR CD34+CD309 OR CD133+VEGFR2+ OR AC133+VEGFR2+ OR AC133+KDR+ OR CD34+KDR+CD133+ OR CD34+VEGFR2+CD133+ OR CD34+KDR+CD45- OR CD34+KDR+CD45dim
