# Supplementary material for: The impact of different forms of exercise on circulating endothelial progenitor cells in cardiovascular and metabolic disease
Source: Eur J Appl Physiol. 2022 Jan 12;122(4):815–60. doi: 10.1007/s00421-021-04876-1 (PMC8927049; doi:10.1007/s00421-021-04876-1)
Supplement: Supplementary file 2 — Supplementary file2 (DOCX 18 KB) [file 421_2021_4876_MOESM2_ESM.docx]

**S2. Full text articles reviewed excluded with reason**

| **Study** | | | **Reason for exclusion** | |
| --- | --- | --- | --- | --- |
| 1. Adams et al., 2013 | | | Adiponectin induced circulating angiogenic cell migration | |
| 1. Aoki et al., 2012 | | | CD34^+^/CD133^+^ cells not EPCs | |
| 1. Barcellos et al., 2012 | | | Published protocol | |
| 1. Bittencourt et al., 2017 | | | No structured exercise | |
| 1. Bonsignore et al., 2002 | | | Measurement of hematopoietic progenitor cells not EPCs | |
| 1. Brehm et al., 2009 | | | Measurement of circulating progenitor cells, not EPCs | |
| 1. Cesari et al., 2014 | | | Observation/follow up study, no structured exercise | |
| 1. Choi et al., 2014 | | | Cell cultured CFU cells, not circulating EPCs | |
| 1. Ciulla et al., 2005 | | | No structured exercise, case study | |
| 1. Chan et al., 2017 | | | End stage renal disease patients | |
| 1. D’Ascenzi et al., 2015 | | | No baseline data to compare pre vs post | |
| 1. Eleuteri et al., 2011 | | | No EPCs assessment | |
| 1. Goussetis et al., 2009 | | | Cell cultured CFU-Hill cells, not circulating EPCs | |
| 1. Guhanarayan et al., 2014 | | | Detraining assessment | |
| 1. Hammadah et al., 2018 | | | Measurements of circulating progenitor cells, not EPCs | |
| 1. Hoetzer et al., 2007 | | | Cell cultured CFU cells, not circulating EPCs | |
| 1. Huang et al., 2017 | | | Diet intervention | |
| 1. Ikeda et al., 2008 | | | Measurement of circulating progenitor cells not circulating EPCs | |
| 1. Jenkins et al., 2009 | | | Cell cultured CFU-Hill cells, not circulating EPCs | |
| 1. Kourek et al., 2021 | | | Parallel publication, no additional data | |
| 1. Kropfl et al., 2020 | | | Measurement of hematopoietic progenitor cells not EPCs | |
| 1. Kropfl et al., 2021 | | | Measurement of hematopoietic progenitor cells not EPCs | |
| 1. Kruger et al., 2014 | | | Not structured exercise | |
| 1. La Vignera et al., 2011 | | | Erectile dysfunction patients | |
| 1. Landers-Ramos et al., 2015 | | | No circulating EPCs assessment | |
| 1. Liao et al., 2016 | | | End stage renal disease patients | |
| 1. Li et al., 2021 | | | Cell cultured CFU-Hill cells, not circulating EPCs | |
| **S2,** continued | | |  |  |
| **Study** | | | **Reason for exclusion** | |
| 1. Lippincott et al., 2008 | No structured exercise | |  |  |
| 1. Manfredini et al., 2009 | Haemodialysis patients | |  |  |
| 1. Moazzami et al., 2020 | Measurement of hematopoietic progenitor cells not EPCs | |  |  |
| 1. Morici et al., 2005 | Measurement of hematopoietic progenitor cells not EPCs | |  |  |
| 1. Neunhäuserer et al., 2021 | COPD patients | |  |  |
| 1. Niederseer et al., 2016 | Altitude exposure | |  |  |
| 1. Niemiro et al., 2018 | Wheelchair athletes | |  |  |
| 1. Pitha et al., 2015 | Renal transplant recipients | |  |  |
| 1. Rehman et al., 2004 | Heterogeneous population, not pre-specified inclusion criteria | |  |  |
| 1. Rocha et al., 2015 | Inclusion of mental stress | |  |  |
| 1. Rousseau et al., 2010 | No exercise intervention | |  |  |
| 1. Santosa et al., 2016 | CD31^+^ cells not EPCs | |  |  |
| 1. Stefanou et al., 2016 | ICU patients (not independent living individuals) | |  |  |
| 1. Sonnenschein et al., 2011 | Cell cultured early EPCs, not circulating EPCs by flow cytometry | |  |  |
| 1. Stelzer et al., 2015 | Measurement of hematopoietic progenitor cells not EPCs | |  |  |
| 1. Theiss et al., 2008 | Altitude exposure, various circulating progenitor cells assessed but not EPCs | |  |  |
| 1. Thorell et al., 2009 | Cell cultured late outgrowth endothelial cells | |  |  |
| 1. Van Craenenbroeck et al., 2015 | Chronic kidney disease patients with no established cardiovascular disease | |  |  |
| 1. Wang et al., 2014 | Hypoxic training | |  |  |
| 1. Witkowski et al., 2010 | Detraining | |  |  |
| 1. Zhao et al., 2016 | No exercise intervention | |  |  |
| 1. Zhen et al., 2016 | Neuromuscular electrical stimulation and physical therapy | |  |  |
| *EPCs* endothelial progenitor cells, *CFU* colony forming units, *COPD* chronic obstructive pulmonary disease, *ICU* intensive care unit. | | |  |  |
