# Supplementary material for: The impact of different forms of exercise on circulating endothelial progenitor cells in cardiovascular and metabolic disease
Source: Eur J Appl Physiol. 2022 Jan 12;122(4):815–60. doi: 10.1007/s00421-021-04876-1 (PMC8927049; doi:10.1007/s00421-021-04876-1)
Supplement: Supplementary file 3 — Supplementary file3 (DOCX 18 KB) [file 421_2021_4876_MOESM3_ESM.docx]

**Supplementary Table 3. Summary of trials that measured number and/or function of cultured MACs**

| **Acute trials** | **Cultured MACs phenotype** | **Results** |
| --- | --- | --- |
| (Adams et al. 2004)  EX group 1: ischaemic CAD, EX group 2: non-ischaemic CAD , EX group 3: healthy. | Di-acLDL^+^/lectin^+^ cells | EX group 1: ↑ 2.9±0.4-fold vs pre-exercise, *P* = 0.001, ↑ 3.3±0.5-fold vs pre-exercise, *P* = 0.001. ↔ changes observed in EX groups 2 and 3 respectively. |
| (Van Craenenbroeck et al. 2009)  EX group 1: CHF type D personality, EX group 2: CHF. | Di-acLDL^+^/lectin^+^ cells | ↑ Migratory capacity of MACs in both groups (*P* = 0.049). No statistical difference between them. |
| (Van Craenenbroeck et al. 2010a)  EX group 1: severe CHF, EX group 2: mild CHF, EX group 3: healthy. | Di-acLDL^+^/lectin^+^ cells | ↑ Migratory capacity of MACs by 52% in EX group 1 (*P =* 0.005*)* and 31% in EX group 2 (*P* = 0.003) and ↓ in EX healthy group 3 ( *P* = 0.04). Post EX MACs levels no different between the three groups. |
| (Sandri et al. 2011)  EX group: stable PAOD. | Di-acLDL^+^/lectin^+^ cells | ↑ MACs by 230.6% (*P* = 0.001). |
| **Chronic trials** | **Cultured MACs phenotype** | **Results** |
| (Erbs et al. 2010)  EX group: CHF , Control group: CHF patients. | MACs | ↑ Migratory capacity by 107.1% compared to control group (*P* < 0.001). |
| (Sandri et al. 2005)  EX group: stable ischaemic PAOD, Control group: stable PAOD. | Di-acLDL^+^/lectin^+^ cells | ↑MACs in EX group, ↑ Integrative capacity (capacity of MACs to participate in network formation) in the EX group (*P* < 0.05), whereas ↔ observed in the controls. |
| (Sandri et al. 2005)  EX group: non-ischaemic prior PAOD, Control group: non-ischaemic prior PAOD. | Di-acLDL^+^/lectin^+^ cells | ↔ MACs number (both groups), ↑ Integrative capacity (EX group). |
| (Sandri et al. 2005)  EX group: stable CAD patients, Control group: stable CAD patients. | Di-acLDL^+^/lectin^+^ cells | ↔ MACs number (both groups), ↑ Integrative capacity (EX group). |
| (Sarto et al. 2007)  EX group: CHF patients. | Di-acLDL^+^/lectin^+^ cells | ↑ MACs by 233%. |
| (Schlager et al. 2011)  EX group: symptomatic PAD, Control group: symptomatic PAD. | Di-acLDL^+^/lectin^+^ cells | ↑ MACs (*P* < 0.05); ↑MACs migratory capacity at 3 and 6 months (*P* < 0.05). |
| (Laufs et al. 2004)  EX group: CAD patients | Di-acLDL^+^/lectin^+^ cells | ↑ MACs numbers; ↓ MACs rate of apoptosis by 41±11%. |
| (Van Craenenbroeck et al. 2010b)  EX group: CHF, Control group: CHF. | Di-acLDL^+^/lectin^+^ cells | ↑ MACs migratory capacity in EX group by 77% (*P* = 0.0001). |
| (Sandri et al. 2016)  EX group ≤55yrs: CHF, EX group ≥65yrs: CHF.  Control group ≤55yrs: CHF, Control group ≥65yrs: CHF | Di-acLDL^+^/lectin^+^ cells | ↑ MACs migratory capacity in EX group. |

**Abbreviations:** *CAD* (coronary artery disease), *CHF* (chronic heart failure), *Di-acLDL* (acetylated Low-Density Lipoprotein labelled with 1,1′-dioctadecyl – 3,3,3′,3′-tetramethyl-indocarbocyanine perchlorate), *EX* (Exercise), *MACs* (Myeloid Angiogenic Cells), *PAOD* (peripheral arterial occlusion disease), ↑ indicates significant increase, ↓ indicates significant decrease, ↔ indicates no significant change.
